# Normal myeloid progenitor cell subset-associated gene signatures for acute myeloid leukaemia subtyping with prognostic impact

**DOI:** 10.1371/journal.pone.0229593

**Published:** 2020-04-23

**Authors:** Anna A. Schönherz, Julie Støve Bødker, Alexander Schmitz, Rasmus Froberg Brøndum, Lasse Hjort Jakobsen, Anne Stidsholt Roug, Marianne T. Severinsen, Tarec C. El-Galaly, Paw Jensen, Hans Erik Johnsen, Martin Bøgsted, Karen Dybkær

**Affiliations:** 1 Department of Clinical Medicine, Faculty of Medicine, Aalborg University, Aalborg, Denmark; 2 Department of Haematology, Clinical Cancer Research Center, Aalborg University Hospital, Aalborg, Denmark; 3 Department of Molecular Biology and Genetics, Center for Quantitative Genetics and Genomics, Aarhus University, Aarhus, Denmark; European Institute of Oncology, ITALY

## Abstract

Acute myeloid leukaemia (AML) is characterised by phenotypic heterogeneity, which we hypothesise is a consequence of deregulated differentiation with transcriptional reminiscence of the normal compartment or cell-of-origin. Here, we propose a classification system based on normal myeloid progenitor cell subset-associated gene signatures (MAGS) for individual assignments of AML subtypes. We generated a MAGS classifier including the progenitor compartments CD34^+^/CD38^-^ for haematopoietic stem cells (HSCs), CD34^+^/CD38^+^/CD45RA^-^ for megakaryocyte-erythroid progenitors (MEPs), and CD34^+^/CD38^+^/CD45RA^+^ for granulocytic-monocytic progenitors (GMPs) using regularised multinomial regression with three discrete outcomes and an elastic net penalty. The regularisation parameters were chosen by cross-validation, and MAGS assignment accuracy was validated in an independent data set (N = 38; accuracy = 0.79) of sorted normal myeloid subpopulations. The prognostic value of MAGS assignment was studied in two clinical cohorts (TCGA: N = 171; GSE6891: N = 520) and had a significant prognostic impact. Furthermore, multivariate Cox regression analysis using the MAGS subtype, FAB subtype, cytogenetics, molecular genetics, and age as explanatory variables showed independent prognostic value. Molecular characterisation of subtypes by differential gene expression analysis, gene set enrichment analysis, and mutation patterns indicated reduced proliferation and overrepresentation of *RUNX1* and *IDH2* mutations in the HSC subtype; increased proliferation and overrepresentation of *CEBPA* mutations in the MEP subtype; and innate immune activation and overrepresentation of *WT1* mutations in the GMP subtype. We present a differentiation-dependent classification system for AML subtypes with distinct pathogenetic and prognostic importance that can help identify candidates poorly responding to combination chemotherapy and potentially guide alternative treatments.

## Introduction

Compelling evidence demonstrates that acute myeloid leukaemia (AML) is of clonal origin and represents the progeny of a single cell that enters leukaemic transformation due to multiple genetic events that impair cell differentiation and apoptosis and invoke uncontrolled cell proliferation. However, the evolution from the first somatic mutation to full-blown AML is not well mapped. The simplest models predict that each newly acquired somatic mutation during oncogenesis confers a selective advantage that drives successive waves of clonal expansion and deregulated differentiation, with the fittest clone becoming dominant at diagnosis and during relapse. [1,2] The understanding of linage-specific progenitor commitment during AML transformation and subsequent clonal evolution is fundamental to the pathogenesis and treatment of AML. We speculate that the genetic abnormality in clinically relevant clones can be traced back to the normal compartment or cell of origin (COO)–as we have previously shown for lymphoid B-cell malignancies [3–6]–and that knowledge of the COO and its deregulation could provide novel molecular and oncogenic insight into AML subtypes.

Myeloid and lymphoid malignancies are particularly well suited for evaluating the cellular origin on malignant transformation due to our understanding of the normal haematopoietic hierarchy and the availability of analytical tools for the examination of phenotypically defined subpopulations at the single-cell level. Recently, we described a procedure to identify and study the gene expression of immunophenotype-based flow-sorted minor subsets. [7] These subsets can be profiled by global gene expression, and using statistical modelling, we were able to define normal subset-specific B-cell-associated gene signatures (BAGS) for assignment and prognostic evaluation in B-cell malignancies. [4,8,9] We found that the diagnostic heterogeneity in multiple myeloma, chronic lymphocytic leukaemia, and diffuse large B-cell lymphoma reflects a link between differentiation and oncogenesis–a contribution often overlooked.

The phenotypic heterogeneity of AML was initially defined by cytology decades ago, when the French-American-British (FAB) Cooperative Group developed a classification system based on morphologic and cytochemical phenotypes associated with normal end stage myelopoiesis. However, as cytogenetics was introduced, the prognostic impact of the FAB subtypes (M0-M7) became unclear. [10–16] Immunophenotyping demonstrated that poorly differentiated AML blasts are deregulated and differentially disrupted early stem or progenitor cells; however, no uniform leukaemic phenotype has been defined by CD34 and CD38 membrane markers. [17] Here, we hypothesise that the phenotypic heterogeneity of AML is a consequence of deregulated differentiation with transcriptional reminiscence of the normal stem or progenitor COO phenotype. We generated myeloid progenitor cell subset-associated gene signatures (MAGS) and assigned subtypes in clinical cohorts to study their prognostic and potential pathogenetic impacts.

## Material and methods

### Data sets

This study is based on data sets already published and publicly available. Sorted normal myeloid data from healthy donors were retrieved from the Gene Expression Omnibus Database (GEO) and are referred to as the GSE63270, [18] GSE42519, [19] GSE19599, [20] GSE17054, [21] and GSE19429 [22] cohorts. For GSE63270, [18] and GSE17054, [21] human bone marrow mononuclear cells (BMMCs) from healthy donors were purchased from ALLCELLS (Emeryville, CA) where collection protocols and donor informed consent are approved by an institutional review board (IRB) in compliance to State and Federal regulations. For GSE42519, [19] GSE19599, [20] and GSE19429 [22] BMMCs from healthy donors were collected and analysed after IRB approval and written informed consent was obtained. The samples were previously phenotyped by multiparametric flow cytometry (MFC) and fluorescence-activated cell sorting (FACS) into myeloid subpopulations, and gene expression profiles (GEPs) were obtained using an Affymetrix Human Genome U13z½3 Plus 2.0 Array GeneChip (Affymetrix, Santa Clara, CA). Sorted normal myeloid data were restricted to those five cohorts due to the limited availability of publicly available data sets with comparable FACS and GEP protocols. The subpopulation compositions and cell sorting details are summarised in S1 and S2 Tables. The myeloid subpopulations of interest for the present study were CD34^+^/CD38^-^ haematopoietic stem cells (HSCs), CD34^+^/CD38^+^/CD45RA^+^ granulocytic-monocytic progenitors (GMPs), and CD34^+^/CD38^+^/CD45RA^-^ megakaryocyte-erythroid progenitors (MEPs).

Clinical AML data were retrieved from the GEO database and The Cancer Genome Atlas (TCGA) database and are referred to as the GSE6891 [23,24] and TCGA [25] cohorts, respectively. For GSE6891, [23,24] and TCGA [25] bone marrow aspirates from AML patients were obtained and analysed after patients provided written informed consent in accordance with the Declaration of Helsinki, and the study was approved by all participating institutional review boards. The data sets were chosen because the GEPs were generated in a comparable manner as those of the sorted normal myeloid data sets, and they contained information on age, gender, FAB subtype, cytogenetic risk score, WBC, disease outcome, and genetic aberrations. The metadata of both cohorts are summarised in the S1 Data (S1 and S3 Tables).

### Statistical analysis

All statistical analyses were performed with R version 3.3.3. [26] The statistical analysis is summarised here; for comprehensive documentation, see the (S1 Data). This study followed the guidelines of omics-directed medicine [27–29] whenever possible. Prior to the statistical analysis, all gene expression data were background corrected and normalised using the Robust Multichip Average (RMA) [30] algorithm and summarised at the gene level using a Brainarray custom CDF for the Affymetrix Human Genome U133 Plus 2.0 GeneChip (version 20.0). The clinical cohorts were RMA normalised per cohort, whereas the normal myeloid cohorts were combined into one meta-cohort prior to RMA normalisation. Six normal myeloid HSC, GMP, and MEP samples were randomly selected and used as a training cohort. The remaining 38 normal myeloid samples were used as a validation cohort.

The MAGS classifier for the HSC, MEP, and GMP subsets was obtained from regularised multinomial regression with the cell type as the discrete outcome and the median-centred GEP of the training cohort as the explanatory variable. The model was fitted with an elastic net penalty. [31] The regularisation parameters were determined by cross-validation, and the parameters with the lowest multinomial deviance were chosen. Genes with non-zero coefficients were defined as predictive. Based on those genes, assignment probabilities were estimated to identify the most likely MAGS subtype of a sample. The prediction accuracy of the MAGS classification was validated in the validation cohort. To compensate for cohort-based technical batch effects, the validation cohort was median centred and scaled to have the same variance as the training cohort. The same procedure was applied to the clinical cohorts followed by cohort-based MAGS subtype assignment according to the subtype with the highest predicted probability score and assigning 15% of the samples with the lowest assignment probability as unclassified (UC). To improve statistical power, clinical cohorts were combined into a clinical meta-cohort following MAGS assignment. For biological characterisation, the GEPs of the clinical meta-cohort were batch corrected using the empirical Bayes approach, ComBat, [32] implemented in the Bioconductor package “*sva*” (version 3.18.0). [33]

Survival analyses were performed using the Kaplan-Meier method, log-rank test statistics, and Cox proportional hazards regression analysis for the individual cohorts and the combined clinical meta-cohort. In addition, the prognostic importance of 13 other explanatory variables (FAB subtype, cytogenetic risk score, *CEBPA* aberrations, *FLT3*-itd aberrations, *FLT3*-tkd aberrations, *IDH1* aberrations, *IDH2* aberrations, *KRAS* aberrations, *NPM1* aberrations, *NRAS* aberrations, white blood cell count (WBC), age, and cohort) was investigated by univariate Cox proportional hazards regression analysis in the TCGA (N = 122), GSE6891 (N = 439), and meta-cohort (N = 561), limited to samples with complete records for all investigated variables (for detailed information, see S1 Data). MAGS was evaluated as an independent explanatory variable by multivariate Cox proportional hazards regression analysis for overall survival in all three cohorts, including explanatory variables tested with prognostic effects in the univariate regression analysis in the respective cohorts. Cytogenetic risk score could not be investigated as a confounding variable in the meta-cohort due to differences in cytogenetic risk group stratification between the two cohorts. In the GSE6891 cohort, cytogenetic risk score stratification was based on cytogenetic abnormalities only, [23] whereas in the TCGA cohort, cytogenetic risk score stratification was based on cytogenetics and molecular genetics. [25,34,35] Instead, MAGS was tested for prognostic impact against the cytogenetic risk scores independently in the two clinical cohorts.

### MAGS validation through biological characterisation

The biological characterisation and identification of biological processes associated with MAGS subtypes were investigated through differential gene expression (DGE) analysis, gene set enrichment analysis, and the identification of subtype-specific mutation patterns for well-documented driver mutations. To increase detection power, analyses were conducted for the meta-cohort, but they were restricted to clinical samples with MAGS assignment probabilities ≥ 0.75, if not stated otherwise.

#### DGE analysis

Was performed in R using the *limma* Bioconductor package (version: 3.26.9). [36,37] To identify MAGS subtype-specific differences, clinical samples assigned to one subtype were compared with the remaining samples (Rest), resulting in the following three comparisons: i) HSC vs. Rest, ii) GMP vs. Rest, and iii) MEP vs. Rest. For summary statistics, p-values were adjusted using the Benjamini and Hochberg algorithm, [38] and genes with p ≤ 0.001 were defined as differentially expressed.

#### Enrichment analysis

Was conducted using two different approaches: a classical Gene Ontology (GO) annotation using Fisher’s exact test to identify over-represented GO terms in differentially expressed genes and a computational gene set enrichment analysis (GSEA) approach developed by the Broad Institute that uses a pre-ranked gene list of all profiled genes (S1 Data). The GSEA was performed using the GSEA desktop application (version 3.0) [39] using 2000 permutations of gene set randomisation and the default settings. Gene sets included in the analysis were selected from the Molecular Signature Database (MSigDB, v6.0) [40] using the *Hallmark*, [39,41] *C2-CP*, [39] and C3-TFT MSigDB collections. [39,42] Gene sets including fewer than 15 or more than 500 genes were excluded from the analysis. Gene sets with an adjusted p ≤ 0.05 and a false discovery rate (FDR) q-value for normalised enrichment scores (ES) ≤ 0.25 were considered significantly enriched. [39]

#### Identification of subtype-specific mutation patterns

Was performed for well-documented driver mutations across seven AML oncogenes (*CEBPA*, *IDH1*, *IDH2*, *FLT3* [including both the *FLT3*-itd and the *FLT3*-tkd aberrations], *NPM1*, *NRAS*, and *KRAS*) in the meta-cohort, irrespective of the MAGS assignment probabilities but limited to samples with recorded mutation information (N = 587: N_GSE6891_ = 457, N_TCGA_ = 130). In addition, a second analysis was conducted for 112 genes associated with AML that had been previously characterised and classified. [43] Mutation records for those genes were extracted from exome-wide somatic mutation data available for a subset of the TCGA cohort (N = 130). [25] Potential associations with MAGS subtypes were investigated for each mutation using Fisher’s exact tests with a significance cut-off level of 0.05.

## Results

### Generation and validation of MAGS

**Fig 1:**
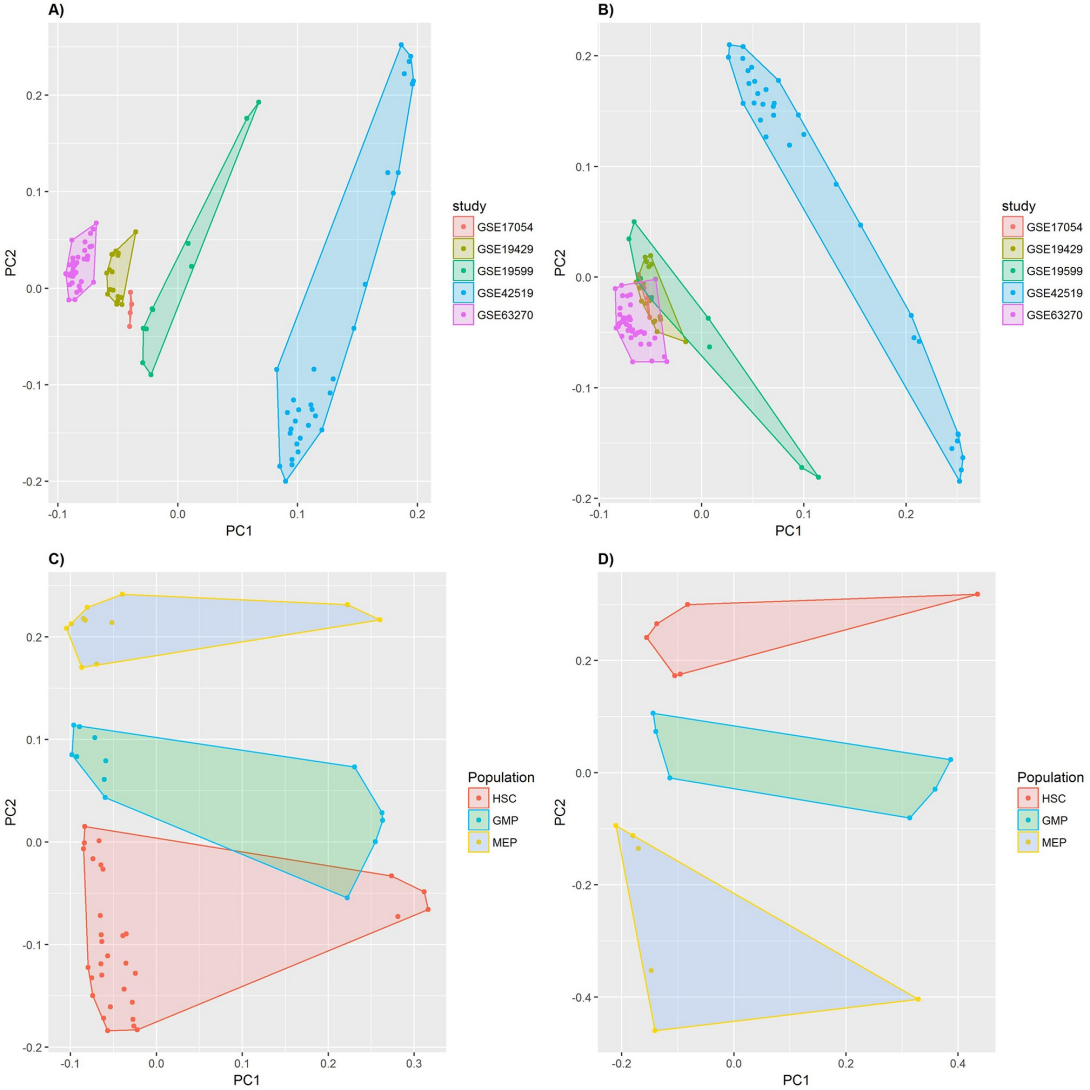
Principal component analysis (PCA) to illustrate variance between samples characterized by gene expression profiles of flow-sorted normal myeloid progenitor subsets. **(A)** The PCA conducted for the meta-cohort of normal myeloid samples that were RMA normalized before combination and includes all myeloid subsets available. **(B)** The PCA conducted for the meta-cohort of normal myeloid samples that were combined prior to RMA normalization and includes all myeloid subsets available. **(C)** The PCA conducted for the meta-cohort described in (B) but limited to samples identified as early (HSC) and late (GMP, MEP) hematopoietic progenitors by FACS. **(D)** The PCA conducted for the meta-cohort described in (B) but limited to samples that were included in the training-cohort and median centered. Each data point represents the expression profile of one sample. In A-B, samples are color-coded based on their cohort identity; for C-D, samples are color-coded according to their myeloid progenitor subset identity (HSC, GMP, MEP). Samples with similar expression profiles will cluster together. Axis labels indicate the principal component (PC) plotted and the proportion of the variance explained by that PC. PCs are derived by orthogonal data transformation to reduce dimensionality and represent the directions of the data that explain a maximal amount of variation.

**Fig 2:**
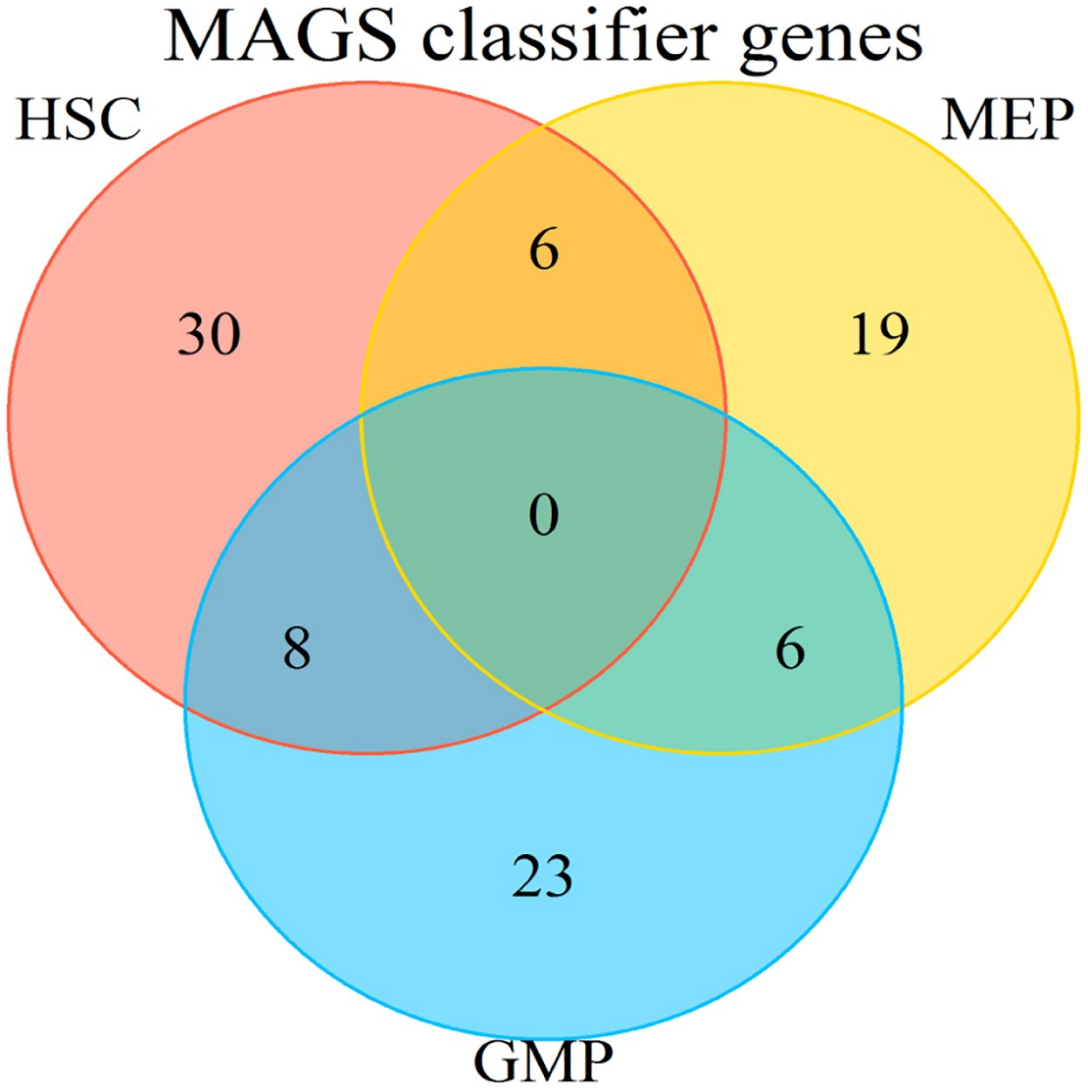
Venn diagram of predictive genes included in the MAGS classification.

The transcriptomic identity of normal myeloid subsets was validated by principal component analysis (Fig 1). Batch effects were partially removed using RMA normalisation (Fig 1A and 1B), and the subset identity could be confirmed for the three progenitor compartments through subset-specific segregation into discrete clusters (Fig 1C and 1D), allowing subsequent identification of MAGS. The MAGS classifier with the smallest deviance determined by cross-validation consisted of 92 genes (S4 Table, S1 Fig). The HSC subtype signature included 44 predictive genes, 30 of which were subtype-specific (68.2%); the GMP subtype signature included 37 predictive genes, 20 of which were subtype-specific (62.2%); and the MEP subtype signature included 33 predictive genes, 19 of which were subtype-specific (57.6%; Fig 2). The highest overlap of predictive genes was between the HSC and GMP subsets (N = 8), followed by GMP and MEP (N = 6) and HSC and MEP (N = 6). The prediction accuracy of the MAGS classifier was validated using sorted normal myeloid samples (N = 38: N_HSC_ = 26, N_GMP_ = 7, N_MEP_ = 5), showing a prediction accuracy of 78.95% when all samples were assigned to one of the three MAGS subtypes and 90.63% when defining 15% of the samples with the lowest MAGS assignment probability as UC. For both assignment strategies, the prediction accuracy of the GMP and MEP subtypes was 100%. The MAGS assignment inconsistencies were restricted to the HSC subtype (Table 1A and 1B). Moreover, the majority of the samples wrongly assigned belonged to the GSE19429 cohort (six of the eight samples).

**Table 1:** MAGS prediction accuracy assigning 100% (A) or 85% (B) of the samples to the defined MAGS subtypes HSC, GMP, MEP, and an additional UC subtype.

**A)**		**Predicted**				**Invalid Prediction (%)**
**Observed**		** HSC (%)**	**MEP (%)**	**GMP (%)**	**UC (%)**	
	**HSC (N = 26)**	18 (69.2)	4 (15.4)	4 (15.4)	**-**	8 (30.8)
	**MEP (N = 5)**	0 (0)	5 (100)	0 (0)	**-**	0 (0)
	**GMP (N = 7)**	0 (0)	0 (0)	7 (100)	**-**	0 (0)
**B)**		**Predicted**				**Invalid Prediction (%)**
**Observed**		**HSC (%)**	**MEP (%)**	**GMP (%)**	**UC (%)**	
	**HSC (N = 26)**	17 (65.4)	3 (11.5)	0 (0)	6 (23.1)	9 (34.6)
	**MEP (N = 5)**	0 (0)	5 (100)	0 (0)	0 (0)	0 (0)
	**GMP (N = 7)**	0 (0)	0 (0)	7 (100)	0 (0)	0 (0)

The prediction accuracy was estimated in the validation-cohort (N = 38). Abbreviations: HSC, hematopoietic stem cells; GMP, granulocytic-monocytic progenitors; MEP, megakaryocyte-erythroid, UC, unclassified.

### MAGS assignment of clinical samples and prognostic impact

Clinical AML samples from two independent cohorts of adult patients diagnosed with *de novo* AML were classified into MAGS subtypes (S3 Table). We allowed 15% of the samples within each cohort to be assigned as UC, resulting in an assignment probability cut-off ≥ 0.71 (TCGA cohort = 0.71, GSE6891 cohort = 0.72). An unambiguous MAGS subtype assignment was achieved, and the subtype frequencies did not vary between the two clinical cohorts (Table 2). Subtype frequencies ranged from 28.1–31.2% in the GSE6981 cohort and 26.4–30.8% in the TCGA cohort when ignoring the UC-assigned samples. Furthermore, the GMP subtype was the most frequently assigned in both cohorts, followed by MEP and HSC.

**Table 2:** Distributions and frequencies of assigned MAGS subtypes across two clinical cohorts: TCGA (N = 182) and GSE6891 (N = 520). Two-sided Fishers exact tests were used to determine significantly different distributions across data sets (p = 0.99).

Cohort	HSC (%)	MEP (%)	GMP (%)	UC (%)	Total (%)
TCGA	50 (27.5)	48 (26.4)	56 (30.8)	28 (15.4)	182 (100)
GSE6891	146 (28.1)	134 (25.8)	162 (31.2)	78 (15.0)	520 (100)

In total, 85% of clinical samples were assigned to MAGS subtypes (HSC, GMP, MEP), and 15% of each cohort was unclassified (UC).

Abbreviations: HSC, hematopoietic stem cells; GMP, granulocytic-monocytic progenitors, MEP, megakaryocyte-erythroid progenitors; UC, unclassified samples (assignment frequency of UC = 15%)

**Fig 3:**
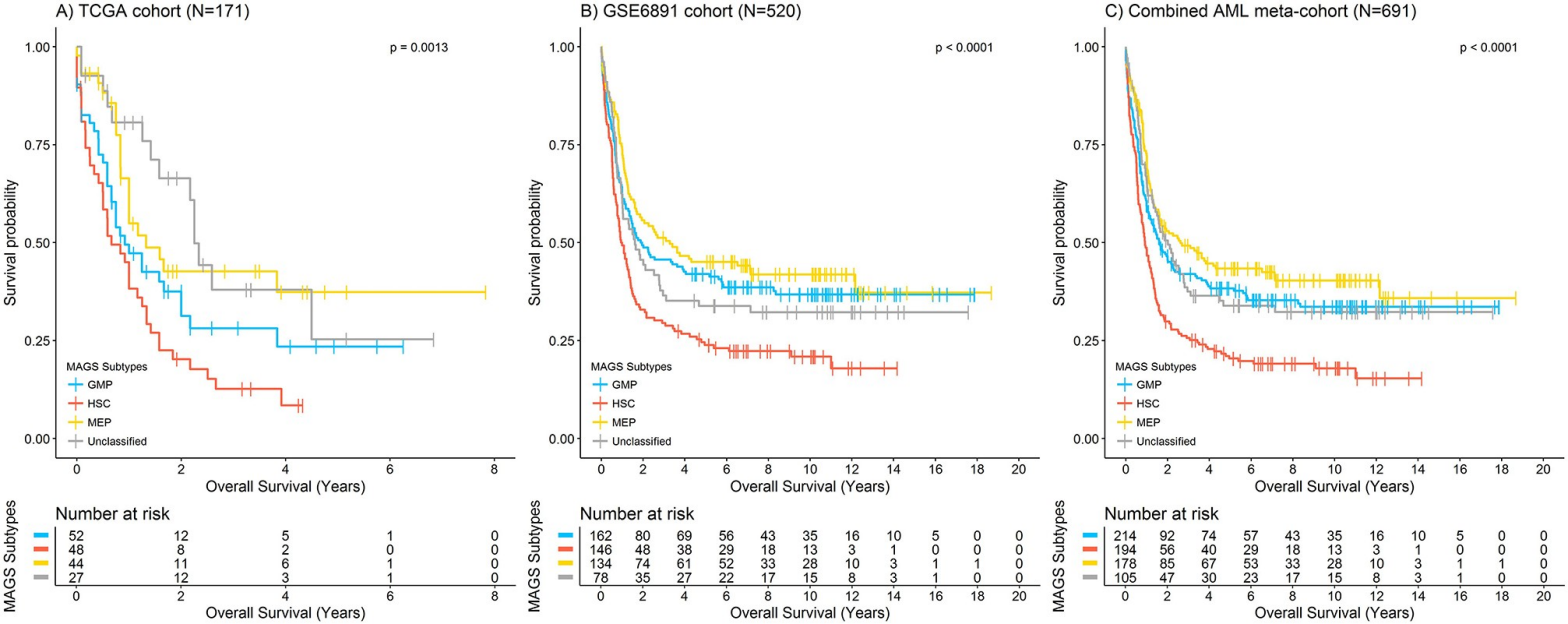
Prognostic validation of the assigned MAGS subtypes for (A) the TCGA-cohort (N = 171), (B) the GSE6892-cohort (N = 520), and (C) the associated meta-cohort (N = 691), using a frequency cut-off of 85% for MAGS assigned and 15% for samples assigned as unclassified. Kaplan Meier survival curves were generated for overall survival and P-values were estimated using a log-rank test. Only samples with complete survival information were included and the number at risk per MAGS subtype are provided for each cohort.

The prognostic impact of the MAGS subtypes was analysed both individually and collectively in a meta-analysis combining the MAGS-assigned samples of the GSE6891 and TCGA cohorts. The MAGS assignment showed a significant prognostic association with overall survival (Fig 3; log-rank test p ≤ 0.001). The lineage-committed MAGS subtypes GMP and MEP had superior prognoses compared with the undifferentiated AMLs captured by the HSC subtype. This was supported by univariate Cox regression analysis conducted for the GSE6891 and the cohort-adjusted clinical meta-cohort, revealing significant differences between the GMP and HSC (GSE6891: HR = 0.63, p < 0.001; meta-cohort: HR = 0.64, p < 0.001), the MEP and HSC (GSE6891: HR = 0.53, p ≪ 0.001; meta-cohort: HR = 0.51, p ≪ 0.001), and the UC and HSC (GSE6891: HR = 0.70, p = 0.03; meta-cohort: HR = 0.60, p < 0.001; Table 3) subtypes. In the TCGA cohort, significant differences were only observed between the MEP and HSC (HR = 0.44, p = 1.9e-03) and the UC and HSC (HR = 0.37, p = 1.6e-03; Table 3) subtypes. Moreover, multivariate Cox proportional hazards analysis conducted for the three cohorts (TCGA, GSE6891, meta-cohort) demonstrated that the MAGS subtypes added significant prognostic information that was not already explained by FAB subtype, cytogenetics, molecular genetics (well-documented driver mutations in *CEBPA*, *FLT3*, *IDH1*, *IDH2*, *KRAS*, *NPM1*, or *NRAS*), or age (Table 4).

**Table 3:** Univariate Cox regression analysis for MAGS-assigned clinical cohorts (GSE6891 cohort: N = 520; TCGA-cohort: N = 171) and the corresponding AML meta-cohort (N = 691) that included both clinical cohorts. Analyses were performed for overall survival. Samples with missing survival information (TCGA cohort: N = 11) were excluded. Table columns indicate the total sample size (N), the number of patients that died (Events) per MAGS subtype, the associated hazards ration (HR), the 95% lower and upper confidence intervals (CI), and the estimated p-value (P).

	N	Events	HR	95% CI	P
**TCGA cohort**					
HSC	48	40	1		
MEP	44	22	0.44	0.26–0.74	1.90e-03**
GMP	52	34	0.68	0.43–1.08	0.1
UC	27	14	0.37	0.20–0.69	1.63e-03**
**GSE6891 cohort**					
HSC	146	115	1		
MEP	134	77	0.53	0.40–0.71	2.13e-05***
GMP	162	101	0.63	0.48–0.83	7.86e-04***
UC	78	52	0.70	0.51–0.98	0.03
**Meta-cohort**^a^					
HSC	194	155	1		
MEP	178	99	0.51	0.40–0.65	1.46e-07***
GMP	214	135	0.64	0.51–0.81	1.64e-04***
UC	105	66	0.60	0.45–0.80	5.32e-04***

^a^ Analysis of the meta-cohort was corrected for potential batch effects by including the cohort (TCGA, GSE6891) as an independent explanatory variable.

Significance levels:

* ≤ 0.05;

** ≤0.01;

*** ≤ 0.001;

Abbreviations: HSC, hematopoietic stem cells; GMP, granulocytic-monocytic progenitors; MEP, megakaryocyte-erythroid progenitors; UC, unclassified samples (assignment frequency of UC = 15%);

**Table 4:** Cox regression analysis of potential confounding variables conducted for the TCGA (N = 122), GSE6891 (N = 439), and meta-cohort (N = 561). Cohorts were limited to samples with complete records for all explanatory variables investigated. Results are shown for the **(A)** univariate Cox regression analysis per explanatory variable and **(B)** associated multivariate Cox regression analyses limited to confounding variables tested significant in univariate Cox regression analyses. Table columns are as described in Table 3. Analyses were performed for overall survival.

**A) Simple Cox Regression**																
	**TCGA cohort (N = 122)**						**GSE6891 cohort (N = 439)**					**AML meta-cohort (N = 561)**				
		**N**	**Events**	**HR**	**95% CI**	**P**	**N**	**Events**	**HR**	**95% CI**	**P**	**N**	**Events**	**HR**	**95% CI**	**P**
**MAGS**	**HSC**	37	32	1			120	93	1			157	125	1		
	**MEP**	26	15	0.523	0.282–0.971	0.040*	108	56	0.491	0.352–0.685	2.75E-05***	134	71	0.486	0.363–0.650	1.26E-06***
	**GMP**	40	26	0.595	0.353–1.005	0.052	147	89	0.632	0.472–0.846	0.002**	187	115	0.622	0.483–0.802	2.50E-04***
	**UC**	19	9	0.309	0.146–0.652	0.002**	64	40	0.666	0.460–0.965	0.032*	83	49	0.580	0.417–0.807	0.001**
**Cytogenetic risk**^a^	**GOOD**	24	10	1			97	40	1			NA	NA	NA	NA	NA
	**INTER**	60	42	2.636	1.319–5.268	0.006**	247	160	1.900	1.341–2.690	3.02E-04***	NA	NA	NA	NA	NA
	**POOR**	35	27	2.828	1.362–5.872	0.005**	85	69	3.222	2.173–4.778	5.84E-09***	NA	NA	NA	NA	NA
	**UC**	3	3	8.159	2.188–30.431	0.002**	10	9	3.801	1.839–7.857	3.14E-04***	NA	NA	NA	NA	NA
**FAB**^b^	**M0**	13	9	1			16	13	1			29	22	1		
	**M1**	28	21	1.773	0.805–3.904	0.155	94	60	0.646	0.355–1.177	0.153	122	81	0.772	0.481–1.236	0.281
	**M2**	27	17	1.156	0.520–2.659	0.697	104	64	0.629	0.346–1.142	0.128	131	81	0.698	0.435–1.119	0.135
	**M3**	6	3	0.542	0.146–2.013	0.360	24	11	0.469	0.210–1.048	0.065	30	14	0.492	0.251–0.962	0.038*
	**M4**	27	16	0.861	0.379–1.955	0.720	79	48	0.635	0.344–1.173	0.147	106	64	0.683	0.420–1.108	0.123
	**M4E**	NA	NA	NA	NA	NA	5	2	0.325	0.073–1.444	0.140	5	2	0.330	0.077–1.403	0.133
	**M5**	14	10	1.957	0.785–4.879	0.150	103	72	0.780	0.432–1.407	0.408	117	82	0.846	0.528–1.356	0.488
	**M6**	3	3	2.452	0.649–9.272	0.186	6	3	0.415	0.118–1.456	0.170	9	6	0.702	0.284–1.731	0.442
	**M7**	3	3	2.311	0.615–8.679	0.215	0	0	NA	NA	NA	3	3	1.920	0.573–6.429	0.290
	**UC**	1	0	2.751	NA	0.996	8	5	0.869	0.310–2.438	0.790	9	5	0.865	0.328–2.286	0.771
**CEBPA**	**NEG**	119	80	1			406	262	1			525	342	1		
	**POS**	3	2	1.184	0.289–4.851	0.815	29	13	0.555	0.318–0.968	0.038*	32	15	0.556	0.332–0.933	0.026*
**FLT3-ITD**	**NEG**	121	81	1			315	187	1			436	268	1		
	**POS**	1	1	5.724	0.766–42.766	0.089	124	91	1.665	1.294–2.141	7.18E-05***	125	92	1.428	1.127–1.811	0.003**
**FLT3-TKD**	**NEG**	107	71	1			390	254	1			497	325	1		
	**POS**	15	11	1.478	0.778–2.810	0.233	48	24	0.699	0.460–1.062	0.093	63	35	0.820	0.579–1.163	0.265
**IDH1**	**NEG**	116	78	1			405	256	1			521	334	1		
	**POS**	6	4	0.772	0.282–2.114	0.614	31	19	0.901	0.565–1.436	0.661	37	23	0.863	0.565–1.316	0.493
**IDH2**	**NEG**	113	78	1			400	254	1			513	332	1		
	**POS**	9	4	0.509	0.186–1.394	0.189	36	21	0.849	0.544–1.324	0.470	45	25	0.765	0.509–1.148	0.196
**KRAS**	**NEG**	116	78	1			436	276	1			552	354	1		
	**POS**	6	4	0.669	0.244–1.836	0.435	3	2	1.028	0.256–4.132	0.970	9	6	1.031	0.460–2.311	0.941
**NMP1**	**NEG**	109	73	1			303	198	1			412	271	1		
	**POS**	13	9	1.739	0.864–3.501	0.121	136	80	0.874	0.674–1.133	0.310	149	89	0.845	0.665–1.074	0.169
**NRAS**	**NEG**	115	77	1			396	253	1			511	330	1		
	**POS**	7	5	1.435	0.578–3.563	0.436	42	25	0.826	0.548–1.246	0.362	49	30	0.843	0.580–1.225	0.370
**WBC**	**WBC**	122	82	1.002	0.998–1.006	0.402	NA	NA	NA	NA	NA	NA	NA	NA	NA	NA
**Age**	**Age**	122	82	1.041	1.022–1.060	1.44E-05***	439	278	1.015	1.005–1.026	0.003**	561	360	1.025	1.017–1.033	2.64E-09***
**Cohort**	**TCGA**	NA	NA	NA	NA	NA	NA	NA	NA	NA	NA	122	82	1		
	**GSE6891**	NA	NA	NA	NA	NA	NA	NA	NA	NA	NA	439	278	0.579	0.451–0.744	1.99E-05***
**B) Multivariate Cox Regression**																
		**TCGA cohort (N = 122)**					**GSE6891 cohort (N = 439)**					**AML meta-cohort (N = 561)**				
		**N**	**Events**	**HR**	**95% CI**	**P**	**N**	**Events**	**HR**	**95% CI**	**P**	**N**	**Events**	**HR**	**95% CI**	**P**
**MAGS**	**HSC**	37	32	1			120	93	1			157	125	1		
	**MEP**	26	15	0.781	0.404–1.511	0.464	108	56	0.600	0.419–0.862	0.006**	134	71	0.524	0.374–0.734	1.69E-04***
	**GMP**	40	26	0.780	0.445–1.365	0.383	147	89	0.723	0.537–0.973	0.032*	187	115	0.593	0.443–0.793	4.32E-04***
	**UC**	19	9	0.467	0.213–1.025	0.058*	64	40	0.742	0.508–1.084	0.123	83	49	0.561	0.398–0.791	9.55E-04***
**Cytogenetic risk**^a^	**GOOD**	24	10	1			97	40	1			-	-	-	-	-
	**INTER**	60	42	1.996	0.964–4.129	0.063	247	160	1.529	1.045–2.239	**0.029**	-	-	-	-	-
	**POOR**	35	27	1.911	0.870–4.195	0.107	85	69	2.751	1.825–4.147	1.36E-06***	-	-	-	-	-
	**UC**	3	3	7.440	1.928–28,712	0.004**	10	9	3.036	1.445–6.379	0.003**	-	-	-	-	-
**FAB**^b^	**M0**	-	-	-	-	-	-	-	-	-	-	29	22	1		
	**M1**	-	-	-	-	-	-	-	-	-	-	122	81	1.267	0.769–2.087	0.353
	**M2**	-	-	-	-	-	-	-	-	-	-	131	81	1.266	0.766–2.094	0.358
	**M3**	-	-	-	-	-	-	-	-	-	-	30	14	1.089	0.531–2.233	0.816
	**M4**	-	-	-	-	-	-	-	-	-	-	106	64	1.143	0.684–1.911	0.610
	**M4E**	-	-	-	-	-	-	-	-	-	-	5	2	0.861	0.196–3.790	0.844
	**M5**	-	-	-	-	-	-	-	-	-	-	117	82	1.595	0.934–2.723	0.087
	**M6**	-	-	-	-	-	-	-	-	-	-	9	6	1.140	0.418–3.112	0.798
	**M7**	-	-	-	-	-	-	-	-	-	-	3	3	2.006	0.573–7.021	0.276
	**UC**	-	-	-	-	-	-	-	-	-	-	9	5	1.186	0.442–3.181	0.735
**CEBPA**	**NEG**	-	-	-	-	-	406	262	1			525	342	1		
	**POS**	-	-	-	-	-	29	13	0.703	0.386–1.283	0.251	32	15	0.759	0.433–1.331	0.336
**FLT3-ITD**	**NEG**	-	-	-	-	-	315	187	1			436	268	1		
	**POS**	-	-	-	-	-	124	91	1.621	1.230–2.135	6.04E-04***	125	92	1.542	1.193–1.994	9.52E-04***
**Age**	**Age**	122	82	1.035	1.017–1.054	1.71E-04***	435	275	1.011	1.001–1.022	0.036*	557	357	1.021	1.012–1.030	4.56E-06***
**Cohort**	**TCGA**	NA	NA	NA	NA	NA	NA	NA	NA	NA	NA	122	82	1		
	**GSE6891**	NA	NA	NA	NA	NA	NA	NA	NA	NA	NA	439	278	0.640	0.472–0.869	0.005**

^a^ Cytogenetic risk was excluded from Cox regression analyses conducted for the meta-cohort due to differences in cytogenetic risk group stratification between the TCGA (stratification based on cytogenetic and molecular genetics) and GSE6891 (stratification based on cytogenetic abnormalities only) cohorts.

^b^ Samples recorded as FAB-Mx (N = 1), FAB-RAEB (N = 4), and FAB-RAEBt (N = 13) were removed.

Significance levels:

* ≤ 0.05;

** ≤0.01;

*** ≤ 0.001;

Abbreviations: N, total sample size; HR, hazards ratio; CI, confidence intervals; HSC, hematopoietic stem cells; MEP, megakaryocyte-erythroid progenitors; GMP, granulocytic- monocytic progenitors; UC, unclassified; INTER, intermediate

### DGE and functional annotation of enriched gene sets

To assess biological differences between MAGS subtypes, we performed DGE analysis on 573 samples and compared each subtype with the combined other subtypes: HSC vs. Rest, GMP vs. Rest, and MEP vs. Rest. The largest number of differentially expressed genes (DEGs) was identified for the GMP subtype (N_DEG_ = 6414), followed by the MEP (N_DEG_ = 4279) and HSC (N_DEG_ = 4071) subtypes. The most distinct DGE profile (number of subtype-specific DEGs) was discovered for the GMP subtype with 1657 DEGs, followed by MEP with 935 and the HSC subtype with 776 (S2 Fig). The top DEGs for the GMP and MEP subtypes overlapped (*HBD*, *ALAS2*, *SPTA1*, *KLF1*, *EPB42*, *AHSP*, and *SELENBP1;* S5A–S5C Table). They were upregulated in the MEP subtype and downregulated in the GMP subtype. Moreover, most of those genes were involved in erythrocyte differentiation (*KLF1*, *ALAS2*, and *AHSP*) or erythrocyte membrane or haemoglobin functions (*HBD*, *SPTA1*, and *EPB42*), which indicates transcriptional discrimination between erythrocytes and other cells. Hence, the results provide biological proof of concept that the MAGS classification of clinical AML samples enables separation into megakaryocyte-erythroid linage and granulocytic-monocytic linage COO subtypes. In contrast, the top DEGs associated with the HSC subtype were subtype-specific and did not reflect any lineage commitment.

To further investigate potential functional patterns associated with the MAGS subtypes, gene enrichment analysis was performed for only the DEGs and taking all genes into consideration. Enrichment analysis of DEGs annotated to GO terms associated with biological processes identified 1066 GO terms enriched for the MEP subtype, followed by 697 for the GMP subtype and 556 for the HSC subtype. Moreover, general patterns of enriched biological processes differed between MAGS subtypes, suggesting different pathogenic courses (S6 Table). The DEGs detected for the HSC subtype were mainly enriched for cell cycle and metabolic processes (S6A Table), whereas DEGs in the GMP and MEP subtypes were mainly enriched for immune system processes and cellular responses to external stimuli, suggesting late progenitor cell functions (S6B and S6C Table).

The GSEA revealed a general downregulation of genes associated with gene sets enriched in the HSC (Table 5A) and MEP subtypes (Table 5B), whereas genes associated with gene sets enriched in the GMP subtype were predominantly upregulated (Table 5C). Biological processes associated with subtype-specific enriched gene sets differed between subtypes. For the HSC subtype, enrichment patterns were characterised by a low cell-cycle activity signature with reduced metabolic rates, suggesting low cell proliferation or a prolonged quiescence phase (S7A Table, S3A Fig). The gene set enrichment patterns observed for the GMP subtype revealed a high metabolic activity signature with innate immune activation (S7B Table, S3B Fig), whereas the MEP subtype had a high cell-cycle activity signature with impaired innate immune activity (S7C Table, S3C Fig). Moreover, the MEP subtype was enriched for genes involved in the metabolism of heme- and erythroblast differentiation, which were downregulated in the GMP subtype, further supporting our hypothesis that malignant cells possess transcriptional reminiscence of the COO.

**Table 5:** Overview of enrichment patterns detected using gene set enrichment analysis (GSEA) in the reduced assignment probability meta-cohort (N = 573: N_GSE6891_ = 423, N_TCGA_ = 150), investigating the *Hallmark*, the *C2-CP*, and the *C3-TFT* gene set collections retrieved from the MSigDB. GSEA analysis was conducted for three comparisons: (A) HSC vs. Rest, (B) MEP vs. Rest, and (C) GMP vs. Rest. The total number of enriched gene sets (Total) with p-value ≤ 0.01 and FDR ≤ 0.25, and the numbers of enriched gene sets detected per group within each comparison are presented.

**A)** *HSC vs*. *Rest*			
**MSigDB collection**	**Total**	**Enriched in HSC (%)**	**Enriched in Rest (%)**
*Hallmark*	31	10 (32.3)	21 (67.7)
*C2-CP*	256	30 (11.7)	226 (88.3)
*C3-TFT*	222	173 (77.9)	49 (22.1)
**B)** *MEP vs*. *Rest*			
**MSigDB collection**	**Total**	**Enriched in GMP (%)**	**Enriched in Rest (%)**
*Hallmark*	25	11 (44.0)	14 (56.0)
*C2-CP*	360	59 (16.4)	301 (83.6)
*C3-TFT*	101	32 (31.7)	69 (68.3)
**C)** G*MP vs*. *Rest*			
**MsigDB collection**	**Total**	**Enriched in MEP (%)**	**Enriched in Rest (%)**
*Hallmark*	21	20 (95.2)	1 (4.8)
*C2-CP*	254	252 (99.2)	301 (0.8)
*C3-TFT*	22	9 (40.9)	69 (59.1)

Abbreviations: HSC, hematopoietic stem cells; MEP, megakaryocyte-erythroid progenitors; GMP, granulocytic-monocytic progenitors; UC, unclassified; MSigDB, Molecular Signature Database

### Annotation of genetic mutation patterns

Potential associations between the MAGS subtypes and well-documented mutations in seven AML-associated oncogenes recorded for both the TCGA and the GSE6891 cohort (*CEBPA*, *FLT3* [including both the *FLT3*-itd and *FLT3*-tkd aberrations], *IDH1*, *IDH2*, *KRAS*, *NPM1*, and *NRAS*) were investigated in the meta-cohort. Two genes, *CEBPA* and *IDH2*, showed subtype-specific mutation patterns. Mutations occurring in the *CEBPA* gene were associated with the MEP subtype (p = 5.79e-08), which was observed especially for the *CEBPA* double mutation. Mutations detected in the *IDH2* gene were more frequently observed in the HSC subtype (p = 0.015; S8A Table). Furthermore, a set of 112 genes previously shown to harbour AML driver mutations were investigated for MAGS subtype-specific mutation patterns in the reduced TCGA cohort (N = 130). Mutations were detected in 68 genes, revealing significant subtype-specific mutation patterns for *RUNX1*, *RUNX1T1*, *TP53*, and *WT1*. *RUNX1* mutations were associated with the HSC subtype (p = 0.005), while mutations detected in the *WT1* gene were associated with the GMP subtype (p = 0.031). Mutations detected in the *TP53* gene were negatively correlated with the GMP subtype, and mutations in *RUNX1T1* were associated with the UC samples (p = 0.02; S8B Table).

## Discussion

In AML, GEPs have successfully identified molecular cancer subtypes for stratifying patients into responders vs. non-responders and predicting survival. [19,44–49] These molecular classification systems are generally based on the GEPs of leukaemic cells or on well-documented oncogenic driver mutations and cytogenetic aberrations associated with AML oncogenesis. [16,21,40–45] Here, we examined and validated a classification system using the GEPs of normal myeloid progenitor cell compartments to classify AML into subtypes based on transcriptional reminiscence of the COO. We showed that the MAGS subtypes of AML cases are associated with prognosis. This observation supports the idea that one or more MAGS subtypes have pathogenic impact. The conclusions may be important for future diagnostic phenotyping and the implementation of individual precision therapy, although there are conceptual, molecular, statistical, and clinical considerations that need to be discussed before clinical implementation and validation.

Our concept is that AML heterogeneity is a consequence of deregulated differentiation and that there is transcriptional reminiscence of the COO. Combining MFC, FACS, and GEP methodologies to phenotype the myeloid progenitor cells in normal bone marrow samples enabled the development of MAGS that differentiate between early (HSC) and late (GMP, MEP) progenitors by tracing transcriptional reminiscence expression patterns of the COO in end-stage AML samples. The MAGS classification assigned comparable subtype frequencies to AML samples within and across independent clinical cohorts. In a meta-analysis of 691 adult patients with *de novo* AML, we demonstrated a significant prognostic association with post-therapy outcome. Moreover, multivariate Cox proportional hazards analyses in the two clinical cohorts as well as in the meta-cohort supported that MAGS subtyping is independent of FAB subtype, cytogenetic risk score (not investigated in the meta-cohort due to different risk score stratifications across cohorts), and molecular genetics (well-documented driver mutations in *CEBPA*, *FLT3* [including both *FLT3*-itd and the *FLT3*-tkd aberrations], *IDH1*, *IDH2*, *KRAS*, *NPM1*, and *NRAS*). These results suggest that we have identified distinct pathogenic mechanisms that require further investigation.

The prognostic impact of MAGS-assigned subtypes conferred a superior prognosis to the lineage-committed GMP and MEP subtypes compared with the undifferentiated HSC subtype. The adverse association between leukaemic stem cell phenotypes and survival is well documented in primary AML. [50,51] In agreement with our findings, those studies demonstrated that leukaemic stem cell signatures were independent prognostic predictors that were associated with adverse clinical outcome.

The molecular characterisation of MAGS subtype-specific mutation patterns revealed that *RUNX1* and *IDH2* mutations had non-random associations with the HSC subtype and that there was a tendency for the *DNMT3A* mutation to be overrepresented in this subtype. Thus, the HSC subtype is associated with well-documented driver mutations in key haematopoietic and epigenetic regulators involved in AML initiation. [25,52,53] In normal haematopoiesis, *RUNX1* plays a critical role in HSC compartment maintenance, proliferation, and haematopoietic differentiation, while in malignancy, genetic aberrations in RUNX1 have been associated with highly aggressive AML and poor prognosis. [43,54–57] In contrast, *IDH2* and *DNMT3A* are key epigenetic regulators, and pre-leukaemic driver mutations in these genes occur early in AML development and are associated with the pre-leukaemic HSC compartment. [2,17,52,58] The significant enrichment of stem cell-specific mutations in AML samples with the HSC subtype indicates a consensus between the transcriptional COO detected by MAGS and the genetic COO, suggesting that AML transformation is not accompanied by subsequent differentiation in the HSC subtype or that HSC-like transcriptional gene signatures were reactivated after AML transformation in more differentiated cells. Similarly, *CEBPA* mutations were overrepresented in the MEP subtype. *CEBPA* is a myeloid transcription factor involved in the balance between cell proliferation and terminal differentiation, especially granulocyte differentiation. Loss of *CEBPA* function in AML contributes to leukaemogenesis by blocking granulocytic differentiation, which is accompanied by the increased accumulation of earlier stem and myeloid progenitors as well as erythroid and megakaryocytic progenitors. [59–62] According to the WHO classification and the current ELN guidelines, patients with mutated CEBPA, particularly those with biallelic mutated CEBPA, represent a cohort with favourable prognosis, [54] which is in general accordance with current findings of superior prognosis in the MEP subtype. However, MAGS-based survival curves revealed smaller differences between subtypes than previously reported findings. [63] Although a positive association between MEP and CEBPA aberrations was observed, this is of associative and not definitive nature, as only 24 out of 147 MEP classified cases showed CEBPA mutations and recorded both contain mono and biallelic CEBPA mutations, possibly contributing to a lower overall survival range than previously reported. [63]

Functional annotation revealed that genes involved in cell-cycle activity and metabolic processes were downregulated in the HSC subtype. This suggests slower cell proliferation or even a prolonged quiescence phase compared with the GMP or MEP subtype. The poor prognosis of the HSC subtype, thus, might reflect the inefficacy of antiproliferative chemotherapeutics, such as cytarabine, in eradicating slow or non-proliferating leukaemic cells. [64,65] Hence, reactivation of cell-cycle activity might be a critical step to re-establish chemotherapy sensitivity, as previously demonstrated in xenograft AML models. [66,67] In addition, the GMP subtype was characterised by enhanced innate immune activity, especially through Toll-like receptor (TLR) signaling, which was impaired in the MEP subtype. Enhanced expression of TLRs has been associated with haematopoietic malignancies, [68–71] including AML, [68,72,73] but their role in pathogenesis remains unclear. Nevertheless, enhanced TLR signaling could activate inflammatory cytokine secretion and downstream effectors, which might explain the observed upregulation of IL-6 JAK-STAT3 signaling and coregulation of TNF-α signaling through NF-κB and IFN-γ signaling in the GMP subtype. Accordingly, inhibition of TLRs or downstream effectors may confer therapeutic benefit in the GMP subtype but not necessarily in the MEP or HSC subtype, as described previously. [68,74]

Statistical models used restricted multinomial regression to estimate the MAGS assignment probability for each sample. MAGS subtypes were defined *a priori* based on FACS and were independent of the GEP used to build the classifier and subsequent MAGS assignment in clinical samples. Furthermore, samples with low assignment probabilities were labelled UC. The frequency of UC samples in other gene expression-based COO classifications is approximately 15%. [4,75] The probability cut-offs observed for MAGS assignment in the clinical cohorts, when allowing for the assignment of 15% of the samples as UC, exceeded 0.70, which is well above the random assignment probability of one out of four. Furthermore, the prognostic robustness of MAGS was successfully validated for a wide range of assignment frequency cut-offs for the UC subtype (S4A–S4C Fig). The prediction accuracy of the MAGS classification was rather low at 78.95%, but defining 15% of the samples with a low assignment probability as UC improved the prediction accuracy to 90.63%. Incorrect subtype prediction was restricted to the HSC subtype, especially to the GSE19429 cohort, for which FACS information was limited. The findings, thus, may be associated with differences in the FACS procedures and poorly defined progenitor populations. This is further supported by recent findings indicating that FACS surface markers are limited in their capacity to fully capture the differentiation stage of haematopoietic progenitor cells. [76] As the prediction accuracy of the MAGS classification is highly dependent on the number and quality of normal myeloid reference populations, it may be improved by increasing the sample size of the training cohort, avoiding interlaboratory batch effects, and optimising the isolation and characterisation of normal haematopoietic cell compartments for *a priori* subtype assignments.

Clinical considerations: Overall, patient survival was associated with the MAGS-assigned AML progenitor subtypes, independent of age, FAB subtype, and cytogenetic risk scores. These findings support the idea that initial hits in oncogenesis occur in the stem and progenitor cell compartments. MAGS subtype-specific mutation patterns of well-documented driver mutations also support the potential clinical impact of MAGS subtyping. Combination chemotherapy still forms the backbone of AML treatments; however, patients with relapsed or refractory diseases have an unmet need for predictive tests and precise companion diagnostics. This need may be fulfilled using MAGS subtyping with predictive information to guide targeted therapy. In agreement with previous work of our group, [4,8] the current analyses indicate that such information is available at diagnosis and could be used for the identification of candidates needing more precise strategies. We believe our results support the future inclusion of gene expression profiling in randomised prospective clinical trials aimed at improving AML treatment.

**In summary**, we have developed and documented a novel classification system that associates normal myeloid progenitor subsets with AML subtypes and prognosis. The MAGS subtypes have different clinical courses, drug resistance mechanisms, and molecular pathogenesis. However, further studies are needed to examine subtype-specific therapeutic strategies. Interestingly, the results imply a consensus between the genetic and transcriptional COOs, suggesting a minor impact of cell plasticity in leukaemic end stage cells. Future prospective studies will be needed to prove this concept using clinical endpoints.

## Supporting information

S1 Data (DOCX)

S1 Table Summary of cohort properties for sorted normal myeloid data sets and clinical AML data sets. (DOCX)

S2 Table FACS antibody panels used to sort early (HSC) and late (GMP, MEP) myeloid progenitor cell subsets. (DOCX)

S3 Table Molecular metadata information available for the GSE6891 and TCGA cohorts. (DOCX)

S4 Table List of predictive genes defining the myeloid progenitor cell subset-associated gene signatures. (DOCX)

S5 Table Top differentially expressed genes. (DOCX)

S6 Table Top twenty Gene Ontology (GO) terms enriched for differentially expressed genes. (DOCX)

S7 Table Top-enriched gene sets identified through GSEA analysis. (DOCX)

S8 Table MAGS subtype-specific mutation patterns. (DOCX)

S9 Table Association between the MAGS and FAB subtype. (DOCX)

S1 Fig Identification of regularisation parameters through cross-validation. (DOCX)

S2 Fig Venn diagram of MAGS subtype-specific differentially expressed genes. (DOCX)

S3 Fig GSEA enrichment plots. (PDF)

S4 Fig Prognostic validation of the assigned MAGS subtypes for the clinical meta-cohort. (DOCX)
