# Supplementary material for: Normal myeloid progenitor cell subset-associated gene signatures for acute myeloid leukaemia subtyping with prognostic impact
Source: PLoS One. 2020 Apr 23;15(4):e0229593. doi: 10.1371/journal.pone.0229593 (PMC7179860; doi:10.1371/journal.pone.0229593)
Supplement: S1 Data — (DOCX) [file pone.0229593.s001.docx]

# **NORMAL MYELOID-PROGENITOR-CELL-SUBSET GENE SIGNATURES FOR ACUTE MYELOID LEUKEMIA ARE ASSOCIATED WITH PROGNOSIS.**

Anna A. Schönherz, Julie Støve Bødker, Alexander Schmitz, Rasmus Froberg Brøndum, Lasse Hjort Jakobsen, Anne Stidsholt Roug, Marianne T. Severinsen, Tarec C. El-Galaly, Paw Jensen, Hans Erik Johnsen, Martin Bøgsted, Karen Dybkær

# **This document contains additional Supplemental Material and Methods, 9 Supplemental Tables and legends, and 4 Supplemental Figures and legends.**

# **Supplemental Material and Methods**

## **Normal sorted myeloid data sets**

CEL files containing gene expression profiles (GEPs) of sorted healthy donor bone marrow samples were retrieved from the Gene Expression Omnibus Database (GEO), at www.ncbi.nlm.nih.gov/geo, and are referred to as the GSE63270, GSE42519, GSE19599, GSE17054, and GSE19429 cohorts. The samples were previously phenotyped by multiparametric flow cytometry (MFC) and fluorescence-activated cell sorting (FACS) into myeloid subpopulations. The GEPs of sorted samples were obtained using the Affymetrix Human Genome U133 Plus2.0 Array GeneChip (Affymetrix, Santa Clara, CA) and deposited as CEL files at the GEO. Normal sorted myeloid data were restricted to those five cohorts due to limited availability of publicly available data sets with comparable FACS- and GEP protocols.

- The **GSE63270 cohort** (N = 20): Normal human bone marrow mononuclear cells from healthy donors were purchased from AllCells Inc. (Catalogue#: ABM006, Emeryville, CA). Mononuclear cells were sorted into hematopoietic stem and progenitor cell populations by flow-cytometry-based cell sorting and the GEP was determined.^1^
- The **GSE42519 cohort** (N = 11): Bone marrow samples were aspirated from the posterior iliac crest of healthy donors, according to the standard protocol of the Department of Hematology, Rigshospitalet, and in accordance with the Declaration of Helsinki. Bone marrow samples were sorted into normal hematopoietic stem and progenitor cell populations, as well as mature myeloid cell subpopulations by FACS and the GEP was determined.^2^
- The **GSE19599 cohort** (N = 4): Bone marrow samples from healthy adult donors were obtained after informed consent, according to the standard protocol of the Department of Hematology, Lund University Hospital, after approval by the Research Ethic Committee of Lund University, Sweden. Bone marrow samples were sorted into hematopoietic progenitor and myeloid cell subpopulations by flow-cytometry-based cell sorting and the GEP was determined.^3^
- The **GSE17054 cohort** (N = 4): Normal human bone marrow mononuclear cells from healthy donors were purchased from AllCells Inc. (Emeryville, CA). Mononuclear cells were sorted into hematopoietic stem cells by flow-cytometry-based cell sorting, using a FACSAria cytometer (BD Biosciences), and the GEP was determined.^4^
- The **GSE19429 cohort** (N = 17): Normal human bone marrow samples from healthy adult donors were obtained after informed consent and approval by the ethics committees (Oxford C00.196, Bournemouth 9991/03/E, Duisburg 2283/03, Stockholm 410/03, Pavia 26264/2002). Mononuclear cells were separated using Histopaque (Sigma-Aldrich, Gillingham, UK) density gradient centrifugation, labeled with CD34 MicroBeads, and then CD34+ cells were isolated using magnetic-activated cell sorting columns (Miltenyi Biotec, Bergisch Gladbach, Germany). The purity of CD34+ cell preparations was evaluated with FACS (>90%).^5^

### **Clinical AML data sets**

Clinical AML data from two publicly available data sets were retrieved from the GEO database and The Cancer Genome Atlas (TCGA) database and are referred to as the GSE6891, and TCGA cohorts, respectively.

- The **GSE6891 cohort** (N = 520): Clinical, cytogenetic, and molecular information and the GEP are available at GEO. Briefly, blood or bone marrow samples of adult patients (<60 years of age) diagnosed with AML were hybridized to the Affymetrix Human Genome U133 Plus2.0 GeneChip as described elsewhere.^6-8^ Patients were newly diagnosed with AML (de novo AML) according to World Health Organization criteria and were treated according to sequential Dutch-Belgian Hemato-Oncology Cooperative Group and the Swiss Group for Clinical Cancer Research (HOVON/SAKK) AML-04, -04A, -29, -32, -42, and -43 protocols^9-11^ (available at http://www.hovon.nl) with no information for individual patients. Cytogenetic risk group stratification is according to HOVON/SAKK and DCOG protocols, only including cytogenetic abnormalities.^12-14^ All patients provided written informed consent in accordance with the Declaration of Helsinki.
- The **TGCA cohort** (N = 182): Clinical, cytogenetic, and molecular information and the GEPs are available through the TCGA legacy archive (https://portal.gdc.cancer.gov/legacy-archive/search/f). The GEPs were generated using the Affymetrix Human Genome U133 Plus2.0 GeneChip (Affymetrix, Santa Clara, CA, USA).^15^ All patients were between ages 18 and 88 and were diagnosed with de novo AML. Patients were treated according to NCCN guidelines (www.nccn.org), with an emphasis on enrollment in therapeutic clinical trials wherever possible. Individual treatment information were available, however, patients were not treated uniformly within the intermediate and unfavorable cytogenetic risk groups.^15^ Cytogenetic risk group stratification is according to the European LeukemiaNet standardized reporting system before 2017 correlating cytogenetic and molecular genetic data.^15-18^

Clinical data sets were chosen because GEPs were generated in a comparable manner as those of normal sorted myeloid data sets and they contained information on disease outcome, age, gender, FAB, cytogenetic risk score, and genetic aberrations. Information for disease outcome (overall survival) was available for 691 samples. Information for disease outcome (overall survival), FAB score, cytogenetic risk score, and age was available for 610 samples, mutation records were available for 587 samples, and complete records for all variables were available for 561 samples (**Tables S3A and S3B**).

## **Data pre-processing of normal myeloid samples**

Normal myeloid cohorts were first combined into one meta-cohort that included all normal samples. Gene expression profiles (GEPs) then were background corrected and normalized using the Robust Multichip Average (RMA)^19^ algorithm, summarized at the gene level using a Brainarray custom CDF for the Affymetrix Human Genome U133 Plus2.0 GeneChip (version 20.0) and median-centered. Normal myeloid cohorts were imbalanced in the number of represented myeloid subsets and sample size per represented subset. A batch correction approach to reduce non-biological batch effects thus could not be applied to avoid removal of biological variation among myeloid subsets. Following normalization, six HSC, GMP, and MEP samples were randomly selected and used as a training-cohort (N_total_ = 18, N_HSC_ = 6, N_GMP_ = 6, N_MEP_ = 6). The remaining samples were used as a validation-cohort (N_total_ = 38, N_HSC_ = 26, N_GMP_ = 7, N_MEP_ = 5).

## **MAGS classification**

The MAGS classifier for the HSC, MEP, and GMP subsets was obtained from regularized multinomial regression with the cell type as the discrete outcome and the median-centered GEP of the training-cohort as the explanatory variable. The model was fitted with an elastic net penalty using the algorithm implemented in the R-package *glmnet* (version 2.0-10).^20^ The elastic net penalty is controlled by two regularization parameters: α and λ. The regularization parameter α can take values between 0 and 1 and thus bridges the gap between the ridge regression penalty (α = 0), allowing all genes in the model, and the Lasso penalty (α = 1), allowing a maximum number of genes equal to the number of samples. The regularization parameter λ is a tuning parameter that determines the overall strength of the penalty and the higher its value the less coefficients will be non-zero. The regularization parameters were determined by cross validation with α ranging from 0.01 to 1 in steps of 0.01, and λ ranging from -14 to 4 (log scale) in steps of 0.045. The adequacies of the models were validated by choosing the minimum multinomial deviance determined by leave-one-sample-out cross-validation. The model with α = 0.38 and log λ = -14 had the smallest cross-validated multinomial deviance (**Figures S1A-B**) leaving us with 92 predictive genes in total (**Table S4**). This indicates, by lowering log λ below -14, that a number of alternative very gene rich models could have described the data well. However, we found the best choice above a suitable compromise between sparsity and robustness. The 92 predictive genes were subsequently used to estimate MAGS subtype assignment probabilities to identify the most likely subtype of a sample.

### **MAGS subtype assignment of AML samples**

Prior to MAGS subtype assignment, all GEPs of the clinical cohorts were RMA normalized for each cohort, separately, and summarized at the gene level using a Brainarray custom CDF for the Affymetrix Human Genome U133 Plus2.0 GeneChip (version 20.0). To compensate for potential technical batch effects between cohorts, each cohort was median centered and scaled at the gene level to have a zero median and the same variance as the training-cohort. Moreover, 15% of the samples with lowest MAGS assignment probability were assigned as unclassified (UC), which was reported for other COO classification systems.^21,22^ Accordingly, unambiguous MAGS subtype assignment was achieved through assignment probabilities ≥ 0.71 (TCGA-cohort = 0.71, GSE6891-cohort = 0.72). The eligibility of an unclassified group was investigated for a wide range of frequency cut-offs (10%, 15%, 20%, 25%), successfully confirming a robust prognostic impact of MAGS (**Figure S4**).

### **Survival analysis**

To investigate the prognostic potential of MAGS, Kaplan-Meier plots, log rank test statistics, as well as simple and multivariate Cox proportional hazards regression analyses were performed using the R-package *survival* (version 2.41-3)^23,24^ and *survminer* (version 0.4.0).^25^ Samples with missing survival information were removed from data cohorts prior to survival analysis. To investigate the prognostic importance of the MAGS classification, Kaplan-Meier plots and univariate Cox proportional hazards regression analysis for overall survival were generated for the entire GSE6891 cohort (N = 520), the reduced TCGA cohort (N = 171), filtered for missing survival information, and the associated meta-cohort (N = 691), combining both clinical cohorts following MAGS assignment. In addition, prognostic importance of cytogenetic risk scores (good, intermediate, poor, unknown) FAB (M0, M1, M2, M3, M4, M4E, M5, M6, M7, unknown) *CEBPA* aberrations (negative, positive), *FLT3*-itd aberrations (negative, positive), *FLT3*-tkd aberrations (negative, positive), *IDH1* aberrations (negative, positive), *IDH2* aberrations (negative, positive), *KRAS* aberrations (negative, positive), *NPM1* aberrations (negative, positive), *NRAS* aberrations (negative, positive), white blood cell count (WBC; continuous), age (continuous), and cohort (TCGA, GSE6891) was evaluated by univariate Cox proportional hazards regression analysis for overall survival in the TCGA cohort (N = 122), the GSE6891 cohort (N = 439), and the associated meta-cohort (N = 561). Cohorts were restricted to samples with complete records for all investigated explanatory variables. Univariate Cox proportional hazards regression analysis with cohort as explanatory variable was investigated only in the meta-cohort. Univariate Cox proportional hazards regression analysis with WBC as explanatory variable was investigated only in the GSE6891 cohort. Univariate Cox proportional hazards regression analysis with cytogenetic risk score as explanatory variable was investigated only in the TCGA and the GSE6891 cohort but not in the meta-cohort due to differences in cytogenetic risk score stratification between cohorts. In the GSE6891 cohort cytogenetic risk score stratification is based on cytogenetic abnormalities only,^6,8^ whereas in the TCGA cohort cytogenetic risk score stratification is based on cytogenetic and molecular genetics.^15-18^ MAGS as an independent explanatory variable was evaluated by multivariate Cox proportional hazards regression analysis for overall survival in the TCGA cohort (N = 122), the GSE6891 cohort (N = 439), and the associated meta-cohort (N = 561) using aforementioned explanatory variables with prognostic effect. The overall model for the multivariate Cox proportional hazards regression analysis in the TCGA cohort included MAGS (HSC, GMP, MEP, UC), cytogenetic risk score (good, intermediate, poor, unknown; stratification based on cytogenetic and molecular genetics), and age (continuous) as potential independent confounders. The overall model for the multivariate Cox proportional hazards regression analysis in the GSE6891 cohort included MAGS (HSC, GMP, MEP, UC), cytogenetic risk score (good, intermediate, poor, unknown; stratification based on cytogenetics only), *CEBPA* (negative, positive), *FLT3*-itd (negative, positive), and age (continuous) as potential independent confounders. The overall model for multivariate Cox proportional hazards regression analysis in the meta-cohort included MAGS (HSC, GMP, MEP, UC), FAB (M0-M7, unknown), *CEBPA* (negative, positive), *FLT3*-itd (negative, positive), age (continuous), and cohort (TCGA, GSE6891) as potential independent confounders. Samples with FAB subtypes other than M0 through M7 (e.g. FAB Mx, RAEB, RAEB-t) were removed from clinical cohorts prior to analysis. The significance level was set to 0.05 and the hazard ratios (HR) were given with 95% confidence intervals.

### **Differential gene expression analysis**

Differential gene expression (DGE) analysis between MAGS subtypes was performed using the bioconducter package *limma* (version: 3.26.9).^26,27^ To increase detection power, DGE analysis was conducted for the meta-cohort but restricted to clinical samples with high MAGS assignment probabilities ≥ 0.75 (N = 573: N_HSC_ = 184, N_GMP_ = 213, N_MEP_ = 176). Briefly, log-fold changes and standard errors were estimated by fitting a linear model for each gene and an empirical Bayes approach was applied to estimate more robust and moderate standard errors.^21^ To identify MAGS subtype-specific differences, clinical samples assigned to one subtype were compared to the remaining samples including the other two subtypes, resulting in the following three comparisons: i) HSC vs. Rest, ii) GMP vs. Rest, and iii) MEP vs. Rest. For summary statistics, p-values were adjusted using the Benjamini and Hochberg procedure^28^ and genes with p-values < 0.001 were defined as differentially expressed. Results for top ranking differentially expressed genes are summarized in **Tables S5A-B**.

### **Enrichment analysis**

Enrichment analysis was performed to investigate potential overrepresentation of specific biological processes or functions associated with the MAGS subtypes. Enrichment analysis was conducted using two different approaches: a classical Gene Ontology (GO) annotation in differentially expressed genes; and a computational gene set enrichment analysis (GSEA) approach developed by the Broad Institute^29^ that uses a pre-ranked gene list of all profiled genes. Enrichment analysis for biological process associated GO terms was performed for differentially expressed genes (see DGE analysis: adjusted p-value < 0.001) using a classical Fisher's exact test implemented in the R-package *topGO* (version 2.22.0).^30^ GO annotation was conducted for the MAGS probability filtered meta-cohort (N = 573: N_HSC_ = 184, N_GMP_ = 213, N_MEP_ = 176), investigating the same contrasts as described for DGE analysis: HSC vs. Rest, GMP vs. Rest, and MEP vs. Rest. Results for GO annotation restricted enrichment analysis are shown in **Tables S6A-C**. Compared with the classical approach, the GSEA approach evaluates whether a priori defined sets of genes (genes associated with specific biological processes) show statistically significant, cumulative changes in gene expression that are correlated with a specific phenotype (MAGS subtypes). To identify MAGS subtype specific enrichment patterns, subtype-specific pre-ranked gene lists were generated and independent enrichment analysis for HSC vs. Rest, GMP vs. Rest, and MEP vs. Rest were conducted using expression data included in the meta-cohort (N = 573: N_HSC_ = 184, N_GMP_ = 213, N_MEP_ = 176). Pre-ranked gene lists were generated by estimating a rank score for each gene to rank all analyzed genes from top up-regulated genes, through non-differentially expressed genes, to top down-regulated genes. Rank scores were estimated based on log-fold changes and p-values generated in the DGE analysis described above using the following rank metric: -log10(p-value) * sign(FC). Subsequently, pre-ranked gene lists were used to assess the gene distribution of each gene set across the ranked list by estimating a running-sum statistic, walking down the ranked list and increasing the running sum when a gene did belong to the set or decreasing it when the gene did not. The extent to which genes in a gene set were overrepresented at the top or bottom of the ranked gene list was calculated through the enrichment score (ES), the maximum deviation from zero encountered in the random walk, and corresponds to a weighted Kolmogorov–Smirnov-like statistic.^31^ The statistical significance of the ES was subsequently estimated using a permutation test, randomly scrambling phenotype labels and re-computing ESs. To compare analysis results across gene sets, ESs were adjusted to account for differences in gene set size and multiple hypothesis testing as described by Subramanian et al.^29^ GSEA was conducted using the GSEA desktop application (version 3.0)^29^ applying 2000 permutations of gene set randomization and default settings otherwise. Gene sets including fewer than 15 or more than 500 genes were excluded from the analysis. Gene sets with an adjusted p-value ≤ 0.05 and a FDR q-value for normalized ES ≤ 0.25 were considered significantly enriched. Gene sets included in the analysis were selected from the Molecular Signature Database (MSigDB)^32^ using the *Hallmark* collection (50 gene sets)^29,33^ as point of departure and the *C2* sub-collection of canonical pathways (*C2*-*CP* collection;( 1329 gene sets)^29^ as well as the *C3* sub-collection of transcription factor targets (*C3-TFT* collection; 615 gene sets)^29,34^ from the MSigDB for in-depth exploration. of associated canonical pathways and cis-regulatory motifs which can function as potential transcription factor binding sites, respectively. The *Hallmark* collection represent specific well-defined biological states or processes with condense information from over 4,000 founder gene sets across all MSigDB collections that based on gene overlap yielded new groups of gene sets with coherent annotation and reduce noise and redundancy. The *C2*-*CP* collection represents pathway gene sets curated from different online pathway databases (e.g. BioCarta, KEGG, Reactome, Pathway Interaction Database, SigmaAldrich) whereas the *C3-TFT* sub-collection represents cis-regulatory motifs which can function as potential transcription factor binding sites. For further details the reader is referred to the Broad Institute GSEA and MSigDB online documentation (<http://software.broadinstitute.org/gsea/msigdb/collections.jsp>).

### **Mutation pattern analysis**

Identification of subtype-specific mutation patterns was performed for two gene sets including previously documented AML driver mutations. One of the sets included 112 genes previously classified as AML driver mutations and used for genomic characterization and AML classification.^35^ The other set included seven well-documented AML oncogenes (*CEBPA, IDH1, IDH2, FLT3, NPM1, NRAS, KRAS*). Records for *FLT3* mutations, furthermore, were separated into *FLT3-*itd mutations, representing in-frame insertions in the JM domain (position aa572 to aa610), and into *FLT3-*tkd mutations, representing point mutations in the second tyrosine kinase domain (position aa738 to aa958), especially at position aa835 and aa836. Mutation records available for the GSE6891 cohort were limited to the seven AML oncogenes and only for a subset of samples (N = 457). For 130 samples of the TCGA cohort, exome sequencing data were generated by the TCGA consortium^15^ and a somatic mutation annotation file (MAF) was publicly available from the TCGA database (TCGA-LAML project). For those samples, mutation records for the 112 genes previously classified as AML driver mutations and the seven well-documented oncogenes recorded in the GSE6891 cohort were extracted. MAGS subtype-associated mutation patterns across the 112 genes were investigated in the reduced TCGA-cohort (N = 130), and MAGS subtype-associated mutation patterns across the seven AML oncogenes were investigated in the meta-cohort, restricted to samples with recorded mutation information (N = 587: N_GSE6891_ = 457, N_TCGA_ = 130). Mutation abundance per MAGS subtype was summarized in contingency tables and potential associations were investigated conducting mutation-wise Fisher´s exact tests with 0.05 as significance cut-off level.

# **Supplemental References**

1. Jung N, Dai B, Gentles AJ, Majeti R, Feinberg AP. An LSC epigenetic signature is largely mutation independent and implicates the HOXA cluster in AML pathogenesis. *Nature Communications.* 2015.

2. Rapin N, Bagger FO, Jendholm J, et al. Comparing cancer vs normal gene expression profiles identifies new disease entities and common transcriptional programs in AML patients. *Blood.* 2014.

3. Andersson A, Eden P, Olofsson T, Fioretos T. Gene expression signatures in childhood acute leukemias are largely unique and distinct from those of normal tissues and other malignancies. *BMC Med Genomics.* 2010;3:6.

4. Majeti R, Becker MW, Tian Q, et al. Dysregulated gene expression networks in human acute myelogenous leukemia stem cells. *Proc Natl Acad Sci U S A.* 2009;106(9):3396-3401.

5. Pellagatti A, Cazzola M, Giagounidis A, et al. Deregulated gene expression pathways in myelodysplastic syndrome hematopoietic stem cells. *Leukemia.* 2010.

6. De Jonge HJM, Valk PJM, Veeger NJGM, et al. High VEGFC expression is associated with unique gene expression profiles and predicts adverse prognosis in pediatric and adult acute myeloid leukemia. *Blood.* 2010.

7. Valk PJM, Verhaak RGW, Beijen MA, et al. Prognostically Useful Gene-Expression Profiles in Acute Myeloid Leukemia. *New England Journal of Medicine.* 2004.

8. Verhaak RGW, Wouters BJ, Erpelinck CAJ, et al. Prediction of molecular subtypes in acute myeloid leukemia based on gene expression profiling. *Haematologica.* 2009.

9. Lowenberg B, Boogaerts MA, Daenen SM, et al. Value of different modalities of granulocyte-macrophage colony-stimulating factor applied during or after induction therapy of acute myeloid leukemia. *J Clin Oncol.* 1997;15(12):3496-3506.

10. Lowenberg B, van Putten W, Theobald M, et al. Effect of priming with granulocyte colony-stimulating factor on the outcome of chemotherapy for acute myeloid leukemia. *N Engl J Med.* 2003;349(8):743-752.

11. Ossenkoppele GJ, Graveland WJ, Sonneveld P, et al. The value of fludarabine in addition to ARA-C and G-CSF in the treatment of patients with high-risk myelodysplastic syndromes and AML in elderly patients. *Blood.* 2004;103(8):2908-2913.

12. Cornelissen JJ, van Putten WLJ, Verdonck LF, et al. Results of a HOVON/SAKK donor versus no-donor analysis of myeloablative HLA-identical sibling stem cell transplantation in first remission acute myeloid leukemia in young and middle-aged adults: benefits for whom? *Blood.* 2007;109(9):3658-3666.

13. Grimwade D, Walker H, Oliver F, et al. The importance of diagnostic cytogenetics on outcome in AML: analysis of 1,612 patients entered into the MRC AML 10 trial. The Medical Research Council Adult and Children's Leukaemia Working Parties. *Blood.* 1998;92(7):2322-2333.

14. van der Holt B, Breems DA, Beverloo HB, et al. Various distinctive cytogenetic abnormalities in patients with acute myeloid leukaemia aged 60 years and older express adverse prognostic value: results from a prospective clinical trial. *British Journal of Haematology.* 2007;136(1):96-105.

15. Ley TJ, Miller C, Ding L, et al. Genomic and epigenomic landscapes of adult de novo acute myeloid leukemia. *The New England journal of medicine.* 2013.

16. Dohner H, Estey EH, Amadori S, et al. Diagnosis and management of acute myeloid leukemia in adults: recommendations from an international expert panel, on behalf of the European LeukemiaNet. *Blood.* 2010;115(3):453-474.

17. Mrozek K, Marcucci G, Nicolet D, et al. Prognostic significance of the European LeukemiaNet standardized system for reporting cytogenetic and molecular alterations in adults with acute myeloid leukemia. *J Clin Oncol.* 2012;30(36):4515-4523.

18. Patel JP, Gonen M, Figueroa ME, et al. Prognostic relevance of integrated genetic profiling in acute myeloid leukemia. *N Engl J Med.* 2012;366(12):1079-1089.

19. Irizarry RA, Hobbs B, Collin F, et al. Exploration, normalization, and summaries of high density oligonucleotide array probe level data. *Biostatistics.* 2003;4(2):249-264.

20. Friedman J, Hastie T, Tibshirani R. Regularization Paths for Generalized Linear Models via Coordinate Descent. *Journal of Statistical Software.* 2010.

21. Dybkær K, Bøgsted M, Falgreen S, et al. Diffuse large B-cell lymphoma classification system that associates normal B-cell subset phenotypes with prognosis. *Journal of Clinical Oncology.* 2015.

22. Lenz G, Wright G, Dave SS, et al. Stromal gene signatures in large-B-cell lymphomas. *N Engl J Med.* 2008;359(22):2313-2323.

23. Therneau TM. A Package for Survival Analysis in S. <https://CRAN.R-project.org/package=survival>. Published 2015. Accessed.

24. Therneau TM, Grambsch PM. *Modeling Survival Data: Extending the Cox Model.* New York: Springer; 2000.

25. Kassambara A, Kosinski M, Biecek P, Scheipl F. survminer: Drawing Survival Curves using “ggplot2” <http://www.sthda.com/english/rpkgs/survminer/>. Published 2017. Accessed.

26. Phipson B, Lee S, Majewski IJ, Alexander WS, Smyth GK. Robust hyperparameter estimation protects against hypervariable genes and improves power to detect differential expression. *Annals of Applied Statistics.* 2016.

27. Ritchie ME, Phipson B, Wu D, et al. limma powers differential expression analyses for RNA-sequencing and microarray studies. *Nucleic Acids Res.* 2015;43(7):e47.

28. Benjamini Y, Hochberg Y. Controlling the false discovery rate: a practical and powerful approach to multiple testing. *Journal of the Royal Statistical Society Series B (Methodological).* 1995.

29. Subramanian A, Tamayo P, Mootha VK, et al. Gene set enrichment analysis: A knowledge-based approach for interpreting genome-wide expression profiles. *Proceedings of the National Academy of Sciences.* 2005.

30. Alexa A, Rahnenfuhrer J. topGO: Enrichment Analysis for Gene Ontology. <http://bioconductor.org/packages/release/bioc/html/topGO.html>. Published 2016. Accessed.

31. Hollander M, Wolfe DA, Chicken E. *Nonparametric Statistical Methods.* 2015.

32. Liberzon A, Subramanian A, Pinchback R, Thorvaldsdóttir H, Tamayo P, Mesirov JP. Molecular signatures database (MSigDB) 3.0. *Bioinformatics.* 2011.

33. Liberzon A, Birger C, Thorvaldsdottir H, Ghandi M, Mesirov JP, Tamayo P. The Molecular Signatures Database (MSigDB) hallmark gene set collection. *Cell Syst.* 2015;1(6):417-425.

34. Xie X, Lu J, Kulbokas EJ, et al. Systematic discovery of regulatory motifs in human promoters and 3' UTRs by comparison of several mammals. *Nature.* 2005;434(7031):338-345.

35. Papaemmanuil E, Gerstung M, Bullinger L, et al. Genomic Classification and Prognosis in Acute Myeloid Leukemia. *New England Journal of Medicine.* 2016.
