# Supplementary material for: Normal myeloid progenitor cell subset-associated gene signatures for acute myeloid leukaemia subtyping with prognostic impact
Source: PLoS One. 2020 Apr 23;15(4):e0229593. doi: 10.1371/journal.pone.0229593 (PMC7179860; doi:10.1371/journal.pone.0229593)
Supplement: S1 Table — (DOCX) [file pone.0229593.s002.docx]

**Supplemental Table S1:** Summary of cohort properties for **A)** normal sorted myeloid data sets and **B)** clinical AML data sets.

| **A)** | | | | | | |
| --- | --- | --- | --- | --- | --- | --- |
| **Cohort** | **N** | **Sample Tissue** | **Subset** | **Analysis** | **Source** | **Reference** |
| GSE63270 | 20 | Mononuclear cells from human bone marrow,  AllCells Inc | HSC (7),  GMP (6),  MEP (7) | Classifier generation: training-cohort (2 HSC, 2 GMP, 3 MEP),  Classifier validation: validation-cohort (5 HSC, 4 GMP, 4 MEP) | GEO,  GSE6891 | Jung et al.^1^ |
| GSE42519 | 11 | Human bone marrow from adult healthy donors | HSC (4),  GMP (5),  MEP (2) | Classifier generation: training-cohort (1 HSC, 3 GMP, 1 MEP),  Classifier validation: validation-cohort (3 HSC, 2 GMP, 1 MEP) | GEO,  GSE42519 | Rapin et al.^2^ |
| GSE19599 | 4 | Human bone marrow from adult healthy donors | GMP (2),  MEP (2) | Classifier generation: training-cohort (1 GMP, 2 MEP),  Classifier validation: validation-cohort (1 GMP) | GEO,  GSE19599 | Anderson et al.^3^ |
| GSE17054 | 4 | Mononuclear cells from human bone marrow,  AllCells Inc | HSC (4) | Classifier generation: training-cohort (1 HSC),  Classifier validation: validation-cohort (3 HSC) | GEO,  GSE17054 | Majeti et al*.*^4^ |
| GSE19429 | 17 | Human bone marrow from adult healthy donors | HSC (17) | Classifier generation: training-cohort (2 HSC),  Classifier validation: validation-cohort (15 HSC) | GEO,  GSE19429 | Pellagatti et al.^5^ |
| **B)** | | | | | | |
| **Cohort** | **N** | **Sample Tissue** | **MAGS** | **Analysis** | **Source** | **Reference** |
| GSE6891 | 520 | Blood and bone marrow from adult patients diagnosed with de-novo AML | HSC (127),  GMP (176),  MEP (139),  UC (78) | Survival analysis KM (N = 520),  Simple Cox regression (N = 520),  Simple Cox regression of explanatory variables other than MAGS (N = 439)^a^,  Multivariate Cox regression  (N = 439)^a^,  DGE analysis (N = 423)^b^,  Enrichment analysis (N = 423)^b^,  GSEA (N = 423)^b^,  Mutation association (N = 457)^c^ | GEO,  GSE6891 | Verhaak et al.^8^, De Jonge et al.^6^ |
| TCGA | 182 | Bone marrow from adult patients diagnosed with  de-novo AML | HSC (46),  GMP (60),  MEP (48),  UC (28) | Survival analysis KM (N = 171)^d^,  Simple Cox regression (N = 171)^d^,  Simple Cox regression of explanatory variables other than MAGS (N = 122)^e^,  Multivariate Cox regression  (N = 122)^e^,  DGE analysis (N = 150)^b^,  Enrichment analysis (N = 150)^b^,  GSEA (N = 150)^b^,  Mutation association (N = 130)^c^ | TCGA, TCGA-LAML^f^ | Ley et al.^15^ |

Abbreviations: N, total sample size; GEO, Gene Expression Omnibus Database; MAGS, myeloid-progenitor-cell-subset associated gene signatures; HSC, hematopoietic stem cells; GMP, granulocytic-monocytic progenitors; MEP, megakaryocyte-erythroid progenitors; UC, unclassified; DGE, Differential gene expression analysis; GSEA, Gene set enrichment analysis;  ^a^ Molecular subtype filtered GSE6891 sub-cohort, only including samples with complete records for FAB, cytogenetic risk score, gene aberrations (*CEBPA*, *FLT3*-itd, *FLT3*-tkd, *IDH1*, *IDH2*, *KRAS*, *NPM1*, *NRAS*), age, and MAGS subtype information and limited to FAB subtypes M0-M7, and unknown (FAB Mx = 1, FAB-RAEB = 4, and FAB-RAEBt = 13 removed). ^b^ Probability filtered clinical (TCGA and GSE6891) sub-cohorts, only including samples that passed the MAGS assignment probability threshold of ≥ 0.75. ^c^ Mutation filtered clinical (TCGA and GSE6891) sub-cohorts, only including samples with recorded mutation information. ^d^ OS filtered TCGA sub-cohort, only including samples with complete survival records. ^e^ OS filtered TCGA sub-cohort, only including samples with complete records for FAB, cytogenetic risk score, gene aberrations (*CEBPA*, *FLT3*-itd, *FLT3*-tkd, *IDH1*, *IDH2*, *KRAS*, *NPM1*, *NRAS*), WBC, age, and MAGS subtype information. ^f^ Microarray gene expression data of the TCGA-LAML project were retrieved from the TCGA GDC Legacy Archive (<https://portal.gdc.cancer.gov/legacy-archive/search/f>).
