# Supplementary material for: Normal myeloid progenitor cell subset-associated gene signatures for acute myeloid leukaemia subtyping with prognostic impact
Source: PLoS One. 2020 Apr 23;15(4):e0229593. doi: 10.1371/journal.pone.0229593 (PMC7179860; doi:10.1371/journal.pone.0229593)
Supplement: S2 Table — (DOCX) [file pone.0229593.s003.docx]

**Supplemental Table S2:** FACS antibody panels used to sort early (HSC) and late (GMP, MEP) myeloid progenitor subsets of normal bone marrow samples.

| **Cohort ID** | **Subset** | **CD34+ MACS^a^** | **HSC** | **GMP** | **MEP** | **Reference** |
| --- | --- | --- | --- | --- | --- | --- |
| GSE63270 | HSC  GMP  MEP | CD34+ enrichment | CD34+, CD38-, CD45RA-, CD90+ | CD34+, CD38+, CD45RA+, D123+ | CD34+, CD38+, CD45RA-, D123- | Jung et al.^1^ |
| GSE42519 | HSC  GMP  MEP | CD34+ enrichment | CD34+, CD38-, CD45RA-, CD90+ (intermediate) | CD34+, CD38+, CD45RA+, D123+ (intermediate) | CD34+, CD38+, CD45RA-, CD123- | Rapin et al.^2^ |
| GSE19599 | GMP  MEP | n.a. | n.a. | CD34+, D45RA+, D123low, CD19-, CD10-, CD110-, | CD34+, CD45RA-, CD123low, CD19-, CD10-, CD110+ | Anderson et al.^3^ |
| GSE17054 | HSC | n.a. | CD34+, CD38-, CD90+ | n.a. | n.a. | Majeti et al.^4^ |
| GSE19429 | HSC | CD34+ enrichment | n.a. | n.a. | n.a. | Pellagatti et al.^5^ |

Abbreviations: HSC, hematopoietic stem cells; GMP, granulocytic-monocytic progenitors; MEP, megakaryocyte-erythroid progenitors; ^a^ CD34+ MACS: CD34+ cell enrichment using magnetic cell sorting separation through CD34 labeling with MicroBeads
