# Supplementary material for: Normal myeloid progenitor cell subset-associated gene signatures for acute myeloid leukaemia subtyping with prognostic impact
Source: PLoS One. 2020 Apr 23;15(4):e0229593. doi: 10.1371/journal.pone.0229593 (PMC7179860; doi:10.1371/journal.pone.0229593)
Supplement: S3 Table — (DOCX) [file pone.0229593.s004.docx]

**Supplemental Table S3:** Clinical and molecular metadata information available for the **A)** GSE6891 cohort, and **B)** TCGA cohort including molecular subtype information for the MAGS, FAB, and cytogenetic risk classification systems. Tables are provided in Appendix 2.
