# Supplementary material for: Normal myeloid progenitor cell subset-associated gene signatures for acute myeloid leukaemia subtyping with prognostic impact
Source: PLoS One. 2020 Apr 23;15(4):e0229593. doi: 10.1371/journal.pone.0229593 (PMC7179860; doi:10.1371/journal.pone.0229593)
Supplement: S4 Table — (DOCX) [file pone.0229593.s005.docx]

**Supplemental Table S4:** List of predictive genes defining the myeloid-progenitor-cell-subset-associated gene signatures (MAGS) classification and estimated classification coefficients for each MAGS subtype. Classification coefficients were obtained from regularized multinomial regression with three discrete outcomes (HSC, MEP, GMP) using an elastic net penalty. Genes marked in bold indicate subtype-specific predictive genes.

| Ensembl Gene ID | HGNC Symbol | HSC | MEP | GMP |
| --- | --- | --- | --- | --- |
| ENSG00000108924 | **HLF** | **0.561** | 0.000 | 0.000 |
| ENSG00000184905 | **TCEAL2** | **0.305** | 0.000 | 0.000 |
| ENSG00000153234 | **NR4A2** | **0.218** | 0.000 | 0.000 |
| ENSG00000138678 | **GPAT3** | **0.217** | 0.000 | 0.000 |
| ENSG00000184226 | **PCDH9** | **0.194** | 0.000 | 0.000 |
| ENSG00000109321 | **AREG** | **0.190** | 0.000 | 0.000 |
| ENSG00000168967 | **PMCHL1** | **0.171** | 0.000 | 0.000 |
| ENSG00000258791 | **LINC00520** | **0.131** | 0.000 | 0.000 |
| ENSG00000163659 | **TIPARP** | **0.122** | 0.000 | 0.000 |
| ENSG00000073756 | **PTGS2** | **0.105** | 0.000 | 0.000 |
| ENSG00000090104 | **RGS1** | **0.100** | 0.000 | 0.000 |
| ENSG00000118503 | **TNFAIP3** | **0.051** | 0.000 | 0.000 |
| ENSG00000138722 | **MMRN1** | **0.048** | 0.000 | 0.000 |
| ENSG00000138669 | **PRKG2** | **0.041** | 0.000 | 0.000 |
| ENSG00000179388 | **EGR3** | **0.033** | 0.000 | 0.000 |
| ENSG00000163737 | **PF4** | **0.026** | 0.000 | 0.000 |
| ENSG00000128218 | **VPREB3** | **0.025** | 0.000 | 0.000 |
| ENSG00000162692 | **VCAM1** | **0.011** | 0.000 | 0.000 |
| ENSG00000136929 | **HEMGN** | **0.007** | 0.000 | 0.000 |
| ENSG00000254303 | **n.a.** | **0.002** | 0.000 | 0.000 |
| ENSG00000137076 | **TLN1** | **-0.019** | 0.000 | 0.000 |
| ENSG00000077984 | **CST7** | **-0.043** | 0.000 | 0.000 |
| ENSG00000164626 | **KCNK5** | **-0.048** | 0.000 | 0.000 |
| ENSG00000099956 | **SMARCB1** | **-0.051** | 0.000 | 0.000 |
| ENSG00000183971 | **NPW** | **-0.065** | 0.000 | 0.000 |
| ENSG00000189068 | **VSTM1** | **-0.072** | 0.000 | 0.000 |
| ENSG00000142453 | **CARM1** | **-0.076** | 0.000 | 0.000 |
| ENSG00000170043 | **TRAPPC1** | **-0.088** | 0.000 | 0.000 |
| ENSG00000123143 | **PKN1** | **-0.120** | 0.000 | 0.000 |
| ENSG00000196415 | **PRTN3** | **-0.131** | 0.000 | 0.000 |
| Predictive genes specific to HSC |  | **30** |  |  |
|  |  |  |  |  |
| Ensembl Gene ID | **HGNC Symbol** | **HSC** | **MEP** | **GMP** |
| ENSG00000105610 | **KLF1** | 0.000 | **0,262** | 0,000 |
| ENSG00000180537 | **RNF182** | 0.000 | **0,140** | 0,000 |
| ENSG00000071575 | **TRIB2** | 0.000 | **0.117** | 0.000 |
| ENSG00000167641 | **PPP1R14A** | 0.000 | **0.105** | 0.000 |
| ENSG00000029534 | **ANK1** | 0.000 | **0.105** | 0.000 |
| ENSG00000137267 | **TUBB2A** | 0.000 | **0.075** | 0.000 |
| ENSG00000166831 | **RBPMS2** | 0.000 | **0.072** | 0.000 |
| ENSG00000148288 | **GBGT1** | 0.000 | **0.060** | 0.000 |
| ENSG00000055118 | **KCNH2** | 0.000 | **0.048** | 0.000 |
| ENSG00000169877 | **AHSP** | 0.000 | **0,042** | 0.000 |
| ENSG00000005961 | **ITGA2B** | 0.000 | **0.026** | 0.000 |
| ENSG00000143416 | **SELENBP1** | 0.000 | **0.006** | 0.000 |
| ENSG00000139278 | **GLIPR1** | 0.000 | **-0.014** | 0.000 |
| ENSG00000132465 | **JCHAIN** | 0.000 | **-0.027** | 0.000 |
| ENSG00000126860 | **EVI2A** | 0.000 | **-0.040** | 0.000 |
| ENSG00000121966 | **CXCR4** | 0.000 | **-0.073** | 0.000 |
| ENSG00000143546 | **S100A8** | 0.000 | **-0.079** | 0.000 |
| ENSG00000122025 | **FLT3** | 0.000 | **-0.146** | 0.000 |
| ENSG00000132274 | **TRIM22** | 0.000 | **-0.149** | 0.000 |
| Predictive genes specific to MEP |  |  | **19** |  |
|  |  |  |  |  |
| Ensembl Gene ID | **HGNC Symbol** | **HSC** | **MEP** | **GMP** |
| ENSG00000242550 | **SERPINB10** | 0.000 | 0.000 | **0.170** |
| ENSG00000163563 | **MNDA** | 0.000 | 0.000 | **0.151** |
| ENSG00000157445 | **CACNA2D3** | 0.000 | 0.000 | **0.131** |
| ENSG00000169252 | **ADRB2** | 0.000 | 0.000 | **0.128** |
| ENSG00000100097 | **LGALS1** | 0.000 | 0.000 | **0.120** |
| ENSG00000121552 | **CSTA** | 0.000 | 0.000 | **0.095** |
| ENSG00000124491 | **F13A1** | 0.000 | 0.000 | **0.091** |
| ENSG00000182718 | **ANXA2** | 0.000 | 0.000 | **0.052** |
| ENSG00000120280 | **CXorf21** | 0.000 | 0.000 | **0.042** |
| ENSG00000255833 | **TIFAB** | 0.000 | 0.000 | **0.040** |
| ENSG00000182578 | **CSF1R** | 0.000 | 0.000 | **0.035** |
| ENSG00000179218 | **CALR** | 0.000 | 0.000 | **0.034** |
| ENSG00000095585 | **BLNK** | 0.000 | 0.000 | **0.032** |
| ENSG00000176076 | **KCNE5** | 0.000 | 0.000 | **0.022** |
| ENSG00000005844 | **ITGAL** | 0.000 | 0.000 | **0.010** |
| ENSG00000103811 | **CTSH** | 0.000 | 0.000 | **0.004** |
| ENSG00000163554 | **SPTA1** | 0.000 | 0.000 | **-0.048** |
| AFFX-r2-Bs-dap-3 | **n.a.** | 0.000 | 0.000 | **-0.052** |
| ENSG00000103316 | **CRYM** | 0.000 | 0.000 | **-0.057** |
| ENSG00000133026 | **MYH10** | 0.000 | 0.000 | **-0.060** |
| ENSG00000109255 | **NMU** | 0.000 | 0.000 | **-0.078** |
| ENSG00000115641 | **FHL2** | 0.000 | 0.000 | **-0.209** |
| ENSG00000244734 | **HBB** | 0.000 | 0.000 | **-0.465** |
| Predictive genes specific to GMP |  |  |  | **23** |
|  |  |  |  |  |
| Ensembl Gene ID | **HGNC Symbol** | **HSC** | **MEP** | **GMP** |
| ENSG00000145708 | CRHBP | 0.659 | -0.132 | 0.000 |
| ENSG00000168209 | DDIT4 | 0.177 | -0.122 | 0.000 |
| ENSG00000081041 | CXCL2 | 0.172 | -0.084 | 0.000 |
| ENSG00000110848 | CD69 | 0.136 | -0.003 | 0.000 |
| ENSG00000169429 | CXCL8 | 0.121 | -0.072 | 0.000 |
| ENSG00000145248 | SLC10A4 | -0.049 | 0.440 | 0.000 |
| Predicted genes overlapping HSC & MEP |  | 6 | 6 |  |
|  |  |  |  |  |
| Ensembl Gene ID | **HGNC Symbol** | **HSC** | **MEP** | **GMP** |
| ENSG00000185022 | MAFF | 0.110 | 0.000 | -0.025 |
| ENSG00000164181 | ELOVL7 | 0.044 | 0.000 | -0.093 |
| ENSG00000005381 | MPO | -0.014 | 0.000 | 0.002 |
| ENSG00000149516 | MS4A3 | -0.038 | 0.000 | 0.167 |
| ENSG00000121211 | MND1 | -0.120 | 0.000 | 0.018 |
| ENSG00000108953 | YWHAE | -0.179 | 0.000 | 0.030 |
| ENSG00000108518 | PFN1 | -0.295 | 0.000 | 0.040 |
| ENSG00000172232 | AZU1 | -0.374 | 0.000 | 0.181 |
| Predicted genes overlapping HSC & GMP |  | 8 |  | 8 |
|  |  |  |  |  |
| Ensembl Gene ID | **HGNC Symbol** | **HSC** | **MEP** | **GMP** |
| ENSG00000119865 | CNRIP1 | 0.000 | 0.185 | -0.356 |
| ENSG00000130208 | APOC1 | 0.000 | 0.156 | -0.066 |
| ENSG00000223609 | HBD | 0.000 | 0.066 | -0.441 |
| ENSG00000197629 | MPEG1 | 0.000 | -0.029 | 0.109 |
| ENSG00000090382 | LYZ | 0.000 | -0.050 | 0.011 |
| ENSG00000140968 | IRF8 | 0.000 | -0.068 | 0.104 |
| Predicted genes overlapping MEP & GMP |  |  | 6 | 6 |

Abbreviations: HSC, hematopoietic stem cells; GMP, granulocytic-monocytic progenitors; MEP, megakaryocyte-erythroid progenitors
