# Supplementary material for: Normal myeloid progenitor cell subset-associated gene signatures for acute myeloid leukaemia subtyping with prognostic impact
Source: PLoS One. 2020 Apr 23;15(4):e0229593. doi: 10.1371/journal.pone.0229593 (PMC7179860; doi:10.1371/journal.pone.0229593)
Supplement: S5 Table — (DOCX) [file pone.0229593.s006.docx]

**Supplemental Table S5:** Top differentially expressed genes detected for **(A)** HSC vs. Rest, **(B)** GMP vs. Rest, and **(C)** MEP vs. Rest comparisons. The table shows the absolute estimate of the log2-fold-change corresponding to each contrast (logFC), the average log2-expression level of each gene across all the arrays and channels in the cohort (AveExp), the moderated t-statistic (t), p-values, as well as Benjamini Hochberg adjusted p-values, and the B-statistic that represents log-odds that the gene is differentially expressed (B). Genes marked in bold indicate top-ten differentially expressed genes identified across MAGS subtypes.

| **A) Top 10 DEGs for HSC vs. Rest** | | | | | | | |
| --- | --- | --- | --- | --- | --- | --- | --- |
| **Ensembl Gene ID** | **Gene Name** | **logFC** | **AveExpr** | **t** | **p-value** | **adjusted p-value** | **B** |
| ENSG00000185624 | P4HB | -0.71 | 10.84 | -13.65 | 6.08E-37 | 1.22E-32 | 72.96 |
| ENSG00000042753 | AP2S1 | -0.42 | 9.81 | -12.72 | 7.70E-33 | 7.73E-29 | 63.68 |
| ENSG00000172232 | AZU1 | -2.19 | 11.05 | -12.56 | 3.75E-32 | 2.29E-28 | 62.13 |
| ENSG00000169397 | RNASE3 | -1.52 | 9.61 | -12.55 | 4.55E-32 | 2.29E-28 | 61.94 |
| ENSG00000165175 | MID1IP1 | -0.55 | 9.37 | -12.52 | 5.74E-32 | 2.30E-28 | 61.71 |
| ENSG00000134324 | LPIN1 | 0.55 | 8.03 | 12.29 | 5.85E-31 | 1.96E-27 | 59.43 |
| ENSG00000128311 | TST | -0.65 | 8.57 | -12.25 | 7.95E-31 | 2.28E-27 | 59.13 |
| ENSG00000169919 | GUSB | -0.57 | 10.49 | -12.17 | 1.74E-30 | 4.38E-27 | 58.36 |
| ENSG00000196730 | DAPK1 | 0.80 | 9.11 | 11.81 | 5.95E-29 | 1.22E-25 | 54.89 |
| ENSG00000023191 | RNH1 | -0.30 | 8.53 | -11.81 | 6.07E-29 | 1.22E-25 | 54.88 |
| **B) Top 10 DEGs for GMP vs. Rest** | | | | | | | |
| ENSG00000223609 | **HBD** | -2.83 | 10.12 | -20.38 | 7.69E-70 | 1.54E-65 | 148.13 |
| ENSG00000158578 | **ALAS2** | -2.62 | 8.62 | -19.13 | 1.81E-63 | 1.82E-59 | 133.60 |
| ENSG00000136929 | HEMGN | -2.23 | 7.44 | -18.50 | 2.84E-60 | 1.90E-56 | 126.31 |
| ENSG00000163554 | **SPTA1** | -1.85 | 7.44 | -18.19 | 1.08E-58 | 5.45E-55 | 122.71 |
| ENSG00000105610 | **KLF1** | -1.67 | 7.33 | -17.97 | 1.36E-57 | 5.46E-54 | 120.20 |
| ENSG00000166947 | **EPB42** | -2.01 | 7.67 | -17.69 | 3.42E-56 | 1.14E-52 | 117.01 |
| ENSG00000244734 | HBB | -2.58 | 12.33 | -17.41 | 8.78E-55 | 2.52E-51 | 113.79 |
| ENSG00000169877 | **AHSP** | -2.11 | 8.15 | -17.39 | 1.10E-54 | 2.75E-51 | 113.57 |
| ENSG00000198053 | SIRPA | 1.01 | 8.34 | 17.23 | 6.33E-54 | 1.41E-50 | 111.84 |
| ENSG00000143416 | **SELENBP1** | -1.98 | 7.37 | -17.13 | 2.07E-53 | 4.16E-50 | 110.66 |
| **C) Top 10 DEGs for MEP vs. Rest** | | | | | | | |
| ENSG00000105610 | **KLF1** | 2.22 | 7.33 | 22.78 | 2.94E-82 | 5.90E-78 | 176.25 |
| ENSG00000223609 | **HBD** | 2.89 | 10.12 | 19.88 | 2.78E-67 | 2.79E-63 | 142.17 |
| ENSG00000055118 | KCNH2 | 1.30 | 7.02 | 19.67 | 3.22E-66 | 1.70E-62 | 139.75 |
| ENSG00000169877 | **AHSP** | 2.50 | 8.15 | 19.67 | 3.38E-66 | 1.70E-62 | 139.70 |
| ENSG00000158578 | **ALAS2** | 2.80 | 8.62 | 19.49 | 2.73E-65 | 1.10E-61 | 137.64 |
| ENSG00000163554 | **SPTA1** | 2.05 | 7.44 | 19.23 | 5.78E-64 | 1.93E-60 | 134.62 |
| ENSG00000166947 | **EPB42** | 2.28 | 7.67 | 19.12 | 2.15E-63 | 6.16E-60 | 133.32 |
| ENSG00000143416 | **SELENBP1** | 2.31 | 7.37 | 19.06 | 4.20E-63 | 1.05E-59 | 132.66 |
| ENSG00000029534 | ANK1 | 1.00 | 6.71 | 18.84 | 5.67E-62 | 1.27E-58 | 130.09 |
| ENSG00000004939 | SLC4A1 | 1.81 | 8.85 | 18.01 | 8.49E-58 | 1.71E-54 | 120.58 |

Analyses were conducted in the assignment probability filtered clinical meta-cohort (N = 483: N_GSE6891_ = 347, N_TCGA_ = 136), restricting analysis to samples that passed the MAGS assignment probability threshold of ≥ 0.75. Abbreviations: HSC, hematopoietic stem cells; GMP, granulocytic-monocytic progenitors; MEP, megakaryocyte-erythroid progenitors
