# Supplementary material for: Normal myeloid progenitor cell subset-associated gene signatures for acute myeloid leukaemia subtyping with prognostic impact
Source: PLoS One. 2020 Apr 23;15(4):e0229593. doi: 10.1371/journal.pone.0229593 (PMC7179860; doi:10.1371/journal.pone.0229593)
Supplement: S6 Table — (DOCX) [file pone.0229593.s007.docx]

**Supplemental Table S6:** Top-twenty Gene Ontology (GO) terms enriched for differentially expressed genes identified for: **(A)** HSC vs. Rest, **(B)** GMP vs. Rest, **(C)** MEP vs. Rest contrasts.

| **A) GO enrichment for DEGs detected in the HSC vs. Rest comparison** | | | | | |
| --- | --- | --- | --- | --- | --- |
| **GO ID** | **Biological Process** | **Anno.^a^** | **Sig.^b^** | **Exp.^c^** | **cFisher** |
| GO:0006996 | Organelle organization | 2880 | 885 | 680.12 | 4.30E-23 |
| GO:0000278 | Mitotic cell cycle | 886 | 328 | 209.23 | 1.60E-20 |
| GO:0007005 | Mitochondrion organization | 558 | 227 | 131.77 | 5.20E-20 |
| GO:1903047 | Mitotic cell cycle process | 815 | 305 | 192.46 | 6.30E-20 |
| GO:0007049 | Cell cycle | 1538 | 506 | 363.20 | 1.00E-18 |
| GO:0070125 | Mitochondrial translational elongation | 76 | 52 | 17.95 | 1.40E-16 |
| GO:0022402 | Cell cycle process | 1213 | 401 | 286.45 | 3.30E-15 |
| GO:0042773 | ATP synthesis coupled electron transport | 62 | 44 | 14.64 | 3.70E-15 |
| GO:0042775 | Mitochondrial ATP synthesis coupled electron transport | 61 | 43 | 14.41 | 1.10E-14 |
| GO:0022900 | Electron transport chain | 77 | 50 | 18.18 | 1.40E-14 |
| GO:0022904 | Respiratory electron transport chain | 75 | 49 | 17.71 | 1.70E-14 |
| GO:0045333 | Cellular respiration | 138 | 74 | 32.59 | 2.10E-14 |
| GO:0070126 | Mitochondrial translational termination | 78 | 50 | 18.42 | 2.90E-14 |
| GO:0006415 | Translational termination | 91 | 55 | 21.49 | 5.90E-14 |
| GO:0044772 | Mitotic cell cycle phase transition | 462 | 179 | 109.10 | 1.20E-13 |
| GO:0046034 | ATP metabolic process | 194 | 92 | 45.81 | 2.80E-13 |
| GO:0034641 | Cellular nitrogen compound metabolic pro... | 5551 | 1490 | 1310.89 | 2.90E-13 |
| GO:0009123 | Nucleoside monophosphate metabolic proce... | 258 | 113 | 60.93 | 4.90E-13 |
| GO:0032543 | Mitochondrial translation | 110 | 61 | 25.98 | 5.80E-13 |
| GO:0000819 | Sister chromatid segregation | 200 | 93 | 47.23 | 8.70E-13 |
| **B) GO enrichment for DEGs detected in the GMP vs. Rest comparison** | | | | | |
| GO:0002376 | Immune system process | 2057 | 973 | 770.09 | 3.00E-23 |
| GO:0002764 | Immune response-regulating signaling pathway | 415 | 246 | 155.37 | 4.90E-20 |
| GO:0050776 | Regulation of immune response | 735 | 391 | 275.16 | 3.60E-19 |
| GO:0006955 | Immune response | 1279 | 627 | 478.82 | 5.50E-19 |
| GO:0002757 | Immune response activating signal transduction | 386 | 228 | 144.51 | 2.10E-18 |
| GO:0002768 | Immune response regulating cell surface receptor signaling pathway | 290 | 181 | 108.57 | 2.90E-18 |
| GO:0002253 | Activation of immune response | 432 | 249 | 161.73 | 4.40E-18 |
| GO:0002682 | Regulation of immune system process | 1135 | 559 | 424.91 | 1.80E-17 |
| GO:0002684 | Positive regulation of immune system process | 792 | 410 | 296.50 | 2.20E-17 |
| GO:0050778 | Positive regulation of immune response | 550 | 299 | 205.91 | 1.50E-16 |
| GO:0002429 | Immune response-activating cell surface receptor signaling pathway | 262 | 163 | 98.09 | 2.20E-16 |
| GO:0016192 | Vesicle-mediated transport | 1265 | 609 | 473.58 | 2.60E-16 |
| GO:0001775 | Cell activation | 793 | 400 | 296.88 | 1.20E-14 |
| GO:0042278 | Purine nucleoside metabolic process | 297 | 173 | 111.19 | 1.70E-13 |
| GO:0046128 | Purine ribonucleoside metabolic process | 294 | 171 | 110.07 | 2.70E-13 |
| GO:0050851 | Antigen receptor-mediated signaling pathway | 177 | 113 | 66.26 | 7.40E-13 |
| GO:0009119 | Ribonucleoside metabolic process | 319 | 181 | 119.43 | 1.20E-12 |
| GO:0009116 | Nucleoside metabolic process | 343 | 192 | 128.41 | 1.40E-12 |
| GO:0009144 | Purine nucleoside triphosphate metabolic process | 220 | 133 | 82.36 | 2.70E-12 |
| GO:0009141 | Nucleoside triphosphate metabolic process | 241 | 143 | 90.22 | 3.10E-12 |
| **C) GO enrichment for DEGs detected in the MEP vs. Rest comparison** | | | | | |
| GO:0002376 | Immune system process | 2057 | 744 | 518.57 | < 1e-30 |
| GO:0006955 | Immune response | 1279 | 481 | 322.43 | 5.00E-25 |
| GO:0001775 | Cell activation | 793 | 315 | 199.91 | 1.60E-20 |
| GO:0002682 | Regulation of immune system process | 1135 | 415 | 286.13 | 5.70E-19 |
| GO:0002684 | Positive regulation of immune system process | 792 | 309 | 199.66 | 8.80E-19 |
| GO:0050776 | Regulation of immune response | 735 | 290 | 185.29 | 1.80E-18 |
| GO:0045321 | Leukocyte activation | 645 | 258 | 162.60 | 2.20E-17 |
| GO:0046649 | Lymphocyte activation | 551 | 223 | 138.91 | 7.90E-16 |
| GO:0007159 | Leukocyte cell-cell adhesion | 439 | 186 | 110.67 | 1.20E-15 |
| GO:0050778 | Positive regulation of immune response | 550 | 222 | 138.65 | 1.30E-15 |
| GO:0002252 | Immune effector process | 582 | 232 | 146.72 | 1.50E-15 |
| GO:0006950 | Response to stress | 3169 | 968 | 798.90 | 6.00E-15 |
| GO:0007049 | Cell cycle | 1538 | 514 | 387.73 | 1.30E-14 |
| GO:0002764 | Immune response-regulating signaling pathway | 415 | 174 | 104.62 | 3.20E-14 |
| GO:0006952 | Defense response | 1251 | 429 | 315.38 | 3.50E-14 |
| GO:0002253 | Activation of immune response | 432 | 179 | 108.91 | 4.90E-14 |
| GO:0070486 | Leukocyte aggregation | 408 | 171 | 102.86 | 5.50E-14 |
| GO:0042110 | T cell activation | 400 | 168 | 100.84 | 7.40E-14 |
| GO:0070489 | T cell aggregation | 400 | 168 | 100.84 | 7.40E-14 |
| GO:1903047 | Mitotic cell cycle process | 815 | 298 | 205.46 | 8.70E-14 |

GO annotation was conducted for GO terms associated with biological processes. Analyses were conducted in the assignment probability filtered clinical meta-cohort (N = 483: N_GSE6891_ = 347, N_TCGA_ = 136), restricting analysis to samples that passed the MAGS assignment probability threshold of ≥ 0.75 and samples assigned as unclassified were omitted. Two-sided Fisher´s exact tests were conducted to investigate enrichment of differentially expressed genes. Abbreviations: HSC, hematopoietic stem cells; GMP, granulocytic-monocytic progenitors; MEP, megakaryocyte-erythroid progenitors; ^a^ number of genes annotated to the GO term (herein limited to biological processes); ^b^ number of GO term annotated genes that are differentially expressed; ^c^ number of GO term annotated genes that are expected to be differentially expressed by chance
