# Supplementary material for: Normal myeloid progenitor cell subset-associated gene signatures for acute myeloid leukaemia subtyping with prognostic impact
Source: PLoS One. 2020 Apr 23;15(4):e0229593. doi: 10.1371/journal.pone.0229593 (PMC7179860; doi:10.1371/journal.pone.0229593)
Supplement: S7 Table — (DOCX) [file pone.0229593.s008.docx]

**Supplemental Table S7:** Top-enriched gene sets identified through GSEA analysis for: **(A)** HSC vs. Rest, **(B)** GMP vs. Rest, **(C)** MEP vs. Rest.

| **A) GSEA conducted for HSC vs. Rest** | | | | | | | | | | |  |
| --- | --- | --- | --- | --- | --- | --- | --- | --- | --- | --- | --- |
| **Gene sets enriched in the HSC subtype** | **Source of ref.** | | **Size** | | **ES** | | **NES** | | **NOM p-val** | **FDR**  **q-val** |  |
| TNFA SIGNALING VIA NFKB | Hallmark | | 191 | | 0.62 | | 2.21 | | 0.00 | 0.00 |  |
| INTERFERON ALPHA RESPONSE | Hallmark | | 87 | | 0.56 | | 1.81 | | 0.00 | 0.00 |  |
| UV RESPONSE DN | Hallmark | | 139 | | 0.52 | | 1.80 | | 0.00 | 0.00 |  |
| KRAS SIGNALING UP | Hallmark | | 184 | | 0.49 | | 1.78 | | 0.00 | 0.00 |  |
| INTERFERON GAMMA RESPONSE | Hallmark | | 181 | | 0.42 | | 1.49 | | 0.00 | 0.04 |  |
| TGF BETA SIGNALING | Hallmark | | 52 | | 0.48 | | 1.42 | | 0.03 | 0.06 |  |
| IL2 STAT5 SIGNALING | Hallmark | | 187 | | 0.39 | | 1.39 | | 0.00 | 0.06 |  |
| ANDROGEN RESPONSE | Hallmark | | 92 | | 0.40 | | 1.29 | | 0.05 | 0.12 |  |
| INFLAMMATORY RESPONSE | Hallmark | | 190 | | 0.36 | | 1.28 | | 0.03 | 0.12 |  |
| MYOGENESIS | Hallmark | | 193 | | 0.34 | | 1.22 | | 0.03 | 0.17 |  |
| PAR1 PATHWAY | CP2:BIOCARTA | | 35 | | 0.75 | | 2.05 | | 0.00 | 0.00 |  |
| MYOSIN PATHWAY | CP2:BIOCARTA | | 30 | | 0.72 | | 1.93 | | 0.00 | 0.03 |  |
| SYNTHESIS OF PIPS AT THE PLASMA MEMBRANE | CP2:REACTOME | | 29 | | 0.71 | | 1.91 | | 0.00 | 0.03 |  |
| PHOSPHATIDYLINOSITOL SIGNALING SYSTEM | CP2:KEGG | | 74 | | 0.58 | | 1.84 | | 0.00 | 0.09 |  |
| SIGNALING BY BMP | CP2:REACTOME | | 22 | | 0.72 | | 1.79 | | 0.00 | 0.13 |  |
| GENERIC TRANSCRIPTION PATHWAY | CP2:REACTOME | | 293 | | 0.47 | | 1.78 | | 0.00 | 0.12 |  |
| OLFACTORY SIGNALING PATHWAY | CP2:REACTOME | | 55 | | 0.59 | | 1.76 | | 0.00 | 0.13 |  |
| LONG TERM DEPRESSION | CP2:KEGG | | 65 | | 0.57 | | 1.75 | | 0.00 | 0.13 |  |
| TCR CALCIUM PATHWAY | CP2:PID | | 26 | | 0.67 | | 1.72 | | 0.00 | 0.18 |  |
| VASCULAR SMOOTH MUSCLE CONTRACTION | CP2:KEGG | | 106 | | 0.51 | | 1.71 | | 0.00 | 0.17 |  |
| SPPA PATHWAY | CP2:BIOCARTA | | 20 | | 0.71 | | 1.71 | | 0.01 | 0.15 |  |
| NFAT TFPATHWAY | CP2:PID | | 46 | | 0.59 | | 1.71 | | 0.00 | 0.14 |  |
| CHEMOKINE RECEPTORS BIND CHEMOKINES | CP2:REACTOME | | 45 | | 0.60 | | 1.71 | | 0.01 | 0.14 |  |
| INTEGRIN A4B1 PATHWAY | CP2:PID | | 31 | | 0.64 | | 1.71 | | 0.00 | 0.13 |  |
| NITRIC OXIDE STIMULATES GUANYLATE CYCLASE | CP2:REACTOME | | 23 | | 0.67 | | 1.70 | | 0.00 | 0.13 |  |
| GPCR DOWNSTREAM SIGNALING | CP2:REACTOME | | 480 | | 0.44 | | 1.70 | | 0.00 | 0.13 |  |
| ALK PATHWAY | CP2:BIOCARTA | | 36 | | 0.60 | | 1.67 | | 0.01 | 0.17 |  |
| TASTE TRANSDUCTION | CP2:KEGG | | 34 | | 0.60 | | 1.64 | | 0.01 | 0.21 |  |
| IL27 PATHWAY | CP2:PID | | 26 | | 0.64 | | 1.64 | | 0.01 | 0.20 |  |
| PI METABOLISM | CP2:REACTOME | | 44 | | 0.56 | | 1.62 | | 0.01 | 0.22 |  |
| EN1_01 | C3-TFT | | 102 | | 0.61 | | 2.00 | | 0.00 | 0.00 |  |
| TTANWNANTGGM_UNKNOWN | C3-TFT | | 53 | | 0.64 | | 1.89 | | 0.00 | 0.01 |  |
| OCT1_Q6 | C3-TFT | | 230 | | 0.48 | | 1.74 | | 0.00 | 0.02 |  |
| MZF1_02 | C3-TFT | | 211 | | 0.50 | | 1.79 | | 0.00 | 0.02 |  |
| STAT5A_02 | C3-TFT | | 127 | | 0.51 | | 1.74 | | 0.00 | 0.02 |  |
| RNTCANNRNNYNATTW_UNKNOWN | C3-TFT | | 56 | | 0.59 | | 1.76 | | 0.00 | 0.02 |  |
| FREAC4_01 | C3-TFT | | 134 | | 0.49 | | 1.69 | | 0.00 | 0.02 |  |
| PR_01 | C3-TFT | | 124 | | 0.51 | | 1.71 | | 0.00 | 0.02 |  |
| TTTNNANAGCYR_UNKNOWN | C3-TFT | | 111 | | 0.51 | | 1.70 | | 0.00 | 0.02 |  |
| FOXO1_01 | C3-TFT | | 212 | | 0.46 | | 1.69 | | 0.00 | 0.02 |  |
| OCT1_06 | C3-TFT | | 225 | | 0.46 | | 1.67 | | 0.00 | 0.02 |  |
| RTTTNNNYTGGM_UNKNOWN | C3-TFT | | 139 | | 0.49 | | 1.67 | | 0.00 | 0.02 |  |
| FREAC2_01 | C3-TFT | | 230 | | 0.46 | | 1.66 | | 0.00 | 0.02 |  |
| AR_02 | C3-TFT | | 33 | | 0.62 | | 1.66 | | 0.00 | 0.02 |  |
| LEF1_Q6 | C3-TFT | | 221 | | 0.45 | | 1.65 | | 0.00 | 0.03 |  |
| TATA_C | C3-TFT | | 242 | | 0.45 | | 1.64 | | 0.00 | 0.03 |  |
| IRF7_01 | C3-TFT | | 226 | | 0.45 | | 1.63 | | 0.00 | 0.03 |  |
| HNF1_Q6 | C3-TFT | | 223 | | 0.45 | | 1.60 | | 0.00 | 0.03 |  |
| HP1SITEFACTOR_Q6 | C3-TFT | | 202 | | 0.45 | | 1.61 | | 0.00 | 0.03 |  |
| OCT1_B | C3-TFT | | 231 | | 0.44 | | 1.60 | | 0.00 | 0.03 |  |
| **Gene sets enriched in the Rest (GMP & MEP)** | **Source of ref.** | | **Size** | | **ES** | | **NES** | | **NOM p-val** | **FDR**  **q-val** |  |
| E2F TARGETS | Hallmark | | 185 | | -0.79 | | -2.36 | | 0.00 | 0.00 |  |
| OXIDATIVE PHOSPHORYLATION | Hallmark | | 174 | | -0.80 | | -2.35 | | 0.00 | 0.00 |  |
| MYC TARGETS V1 | Hallmark | | 170 | | -0.74 | | -2.19 | | 0.00 | 0.00 |  |
| G2M CHECKPOINT | Hallmark | | 186 | | -0.71 | | -2.11 | | 0.00 | 0.00 |  |
| PROTEIN SECRETION | Hallmark | | 88 | | -0.71 | | -1.97 | | 0.00 | 0.00 |  |
| MTORC1 SIGNALING | Hallmark | | 185 | | -0.66 | | -1.95 | | 0.00 | 0.00 |  |
| UNFOLDED PROTEIN RESPONSE | Hallmark | | 102 | | -0.69 | | -1.92 | | 0.00 | 0.00 |  |
| FATTY ACID METABOLISM | Hallmark | | 143 | | -0.65 | | -1.89 | | 0.00 | 0.00 |  |
| ADIPOGENESIS | Hallmark | | 182 | | -0.62 | | -1.85 | | 0.00 | 0.00 |  |
| GLYCOLYSIS | Hallmark | | 190 | | -0.61 | | -1.83 | | 0.00 | 0.00 |  |
| DNA REPAIR | Hallmark | | 131 | | -0.63 | | -1.82 | | 0.00 | 0.00 |  |
| HEME METABOLISM | Hallmark | | 189 | | -0.56 | | -1.68 | | 0.00 | 0.00 |  |
| MYC TARGETS V2 | Hallmark | | 55 | | -0.63 | | -1.64 | | 0.00 | 0.00 |  |
| MITOTIC SPINDLE | Hallmark | | 195 | | -0.53 | | -1.58 | | 0.00 | 0.00 |  |
| REACTIVE OXIGEN SPECIES PATHWAY | Hallmark | | 43 | | -0.63 | | -1.58 | | 0.01 | 0.00 |  |
| PEROXISOME | Hallmark | | 97 | | -0.56 | | -1.54 | | 0.00 | 0.01 |  |
| UV RESPONSE UP | Hallmark | | 151 | | -0.52 | | -1.54 | | 0.00 | 0.01 |  |
| ESTROGEN RESPONSE LATE | Hallmark | | 193 | | -0.47 | | -1.39 | | 0.01 | 0.03 |  |
| XENOBIOTIC METABOLISM | Hallmark | | 190 | | -0.46 | | -1.37 | | 0.01 | 0.04 |  |
| PI3K AKT MTOR SIGNALING | Hallmark | | 99 | | -0.48 | | -1.35 | | 0.04 | 0.05 |  |
| DNA REPLICATION | CP2:REACTOME | | 164 | | -0.78 | | -2.31 | | 0.00 | 0.00 |  |
| MITOTIC M M G1 PHASES | CP2:REACTOME | | 147 | | -0.77 | | -2.26 | | 0.00 | 0.00 |  |
| OXIDATIVE PHOSPHORYLATION | CP2:KEGG | | 96 | | -0.81 | | -2.26 | | 0.00 | 0.00 |  |
| CELL CYCLE MITOTIC | CP2:REACTOME | | 273 | | -0.73 | | -2.24 | | 0.00 | 0.00 |  |
| TCA CYCLE AND RESPIRATORY ELECTRON TRANSPORT | CP2:REACTOME | | 96 | | -0.80 | | -2.23 | | 0.00 | 0.00 |  |
| CELL CYCLE CHECKPOINTS | CP2:REACTOME | | 98 | | -0.78 | | -2.21 | | 0.00 | 0.00 |  |
| G1 S TRANSITION | CP2:REACTOME | | 91 | | -0.80 | | -2.20 | | 0.00 | 0.00 |  |
| RESPIRATORY ELECTRON TRANSPORT ATP SYNTHESIS BY CHEMIOSMOTIC COUPLING AND HEAT PRODUCTION BY UNCOUPLING PROTEINS | CP2:REACTOME | | 63 | | -0.83 | | -2.20 | | 0.00 | 0.00 |  |
| PARKINSONS DISEASE | CP2:KEGG | | 95 | | -0.79 | | -2.20 | | 0.00 | 0.00 |  |
| SYNTHESIS OF DNA | CP2:REACTOME | | 78 | | -0.80 | | -2.20 | | 0.00 | 0.00 |  |
| S PHASE | CP2:REACTOME | | 92 | | -0.79 | | -2.19 | | 0.00 | 0.00 |  |
| CELL CYCLE | CP2:REACTOME | | 348 | | -0.70 | | -2.17 | | 0.00 | 0.00 |  |
| RESPIRATORY ELECTRON TRANSPORT | CP2:REACTOME | | 49 | | -0.86 | | -2.17 | | 0.00 | 0.00 |  |
| HUNTINGTONS DISEASE | CP2:KEGG | | 146 | | -0.73 | | -2.14 | | 0.00 | 0.00 |  |
| MITOTIC G1 G1 S PHASES | CP2:REACTOME | | 114 | | -0.74 | | -2.12 | | 0.00 | 0.00 |  |
| M G1 TRANSITION | CP2:REACTOME | | 68 | | -0.79 | | -2.12 | | 0.00 | 0.00 |  |
| APC C CDH1 MEDIATED DEGRADATION OF CDC20 AND OTHER APC C CDH1 TARGETED PROTEINS IN LATE MITOSIS EARLY G1 | CP2:REACTOME | | 53 | | -0.80 | | -2.08 | | 0.00 | 0.00 |  |
| REGULATION OF MITOTIC CELL CYCLE | CP2:REACTOME | | 65 | | -0.77 | | -2.06 | | 0.00 | 0.00 |  |
| ASSEMBLY OF THE PRE REPLICATIVE COMPLEX | CP2:REACTOME | | 54 | | -0.80 | | -2.05 | | 0.00 | 0.00 |  |
| AUTODEGRADATION OF CDH1 BY CDH1 APC C | CP2:REACTOME | | 45 | | -0.80 | | -2.04 | | 0.00 | 0.00 |  |
| SGCGSSAAA_E2F1DP2_01 | C3-TFT | | 149 | | -0.66 | | -1.94 | | 0.00 | 0.00 |  |
| E2F_Q6 | C3-TFT | | 207 | | -0.59 | | -1.78 | | 0.00 | 0.00 |  |
| E2F1_Q6 | C3-TFT | | 210 | | -0.59 | | -1.78 | | 0.00 | 0.00 |  |
| GGAANCGGAANY_UNKNOWN | C3-TFT | | 95 | | -0.64 | | -1.78 | | 0.00 | 0.00 |  |
| CGGAARNGGCNG_UNKNOWN | C3-TFT | | 46 | | -0.69 | | -1.76 | | 0.00 | 0.00 |  |
| E2F_Q6_01 | C3-TFT | | 210 | | -0.58 | | -1.74 | | 0.00 | 0.00 |  |
| ELK1_02 | C3-TFT | | 211 | | -0.58 | | -1.74 | | 0.00 | 0.00 |  |
| E2F1_Q3 | C3-TFT | | 213 | | -0.58 | | -1.74 | | 0.00 | 0.00 |  |
| E2F_Q4 | C3-TFT | | 208 | | -0.57 | | -1.72 | | 0.00 | 0.00 |  |
| E2F4DP2_01 | C3-TFT | | 208 | | -0.57 | | -1.71 | | 0.00 | 0.00 |  |
| E2F_02 | C3-TFT | | 208 | | -0.57 | | -1.70 | | 0.00 | 0.00 |  |
| E2F1DP2_01 | C3-TFT | | 208 | | -0.57 | | -1.70 | | 0.00 | 0.00 |  |
| E2F1DP1_01 | C3-TFT | | 208 | | -0.57 | | -1.69 | | 0.00 | 0.00 |  |
| GABP_B | C3-TFT | | 223 | | -0.56 | | -1.69 | | 0.00 | 0.00 |  |
| E2F4DP1_01 | C3-TFT | | 212 | | -0.56 | | -1.68 | | 0.00 | 0.00 |  |
| E2F1_Q6_01 | C3-TFT | | 213 | | -0.54 | | -1.63 | | 0.00 | 0.00 |  |
| E2F1DP1RB_01 | C3-TFT | | 200 | | -0.54 | | -1.63 | | 0.00 | 0.00 |  |
| E2F_03 | C3-TFT | | 215 | | -0.54 | | -1.63 | | 0.00 | 0.00 |  |
| E2F_Q3_01 | C3-TFT | | 206 | | -0.52 | | -1.58 | | 0.00 | 0.01 |  |
| SREBP1_01 | C3-TFT | | 147 | | -0.54 | | -1.58 | | 0.00 | 0.01 |  |
| **B) GSEA conducted for GMP vs. Rest** | | | | | | | | | | |  |
| **Gene sets enriched in the GMP subtype** | | **Source of ref.** | | **Size** | | **ES** | | **NES** | **NOM p-val** | **FDR**  **q-val** |  |
| OXIDATIVE PHOSPHORYLATION | | Hallmark | | 174 | | 0.73 | | 1.99 | 0.00 | 0.00 |  |
| ADIPOGENESIS | | Hallmark | | 182 | | 0.63 | | 1.71 | 0.00 | 0.00 |  |
| IL6 JAK STAT3 SIGNALING | | Hallmark | | 83 | | 0.66 | | 1.69 | 0.00 | 0.00 |  |
| COMPLEMENT | | Hallmark | | 188 | | 0.61 | | 1.67 | 0.00 | 0.00 |  |
| PROTEIN SECRETION | | Hallmark | | 88 | | 0.65 | | 1.66 | 0.00 | 0.00 |  |
| GLYCOLYSIS | | Hallmark | | 190 | | 0.60 | | 1.62 | 0.00 | 0.00 |  |
| PI3K AKT MTOR SIGNALING | | Hallmark | | 99 | | 0.61 | | 1.57 | 0.00 | 0.01 |  |
| FATTY ACID METABOLISM | | Hallmark | | 143 | | 0.59 | | 1.57 | 0.00 | 0.01 |  |
| PEROXISOME | | Hallmark | | 97 | | 0.60 | | 1.55 | 0.00 | 0.01 |  |
| MTORC1 SIGNALING | | Hallmark | | 185 | | 0.56 | | 1.53 | 0.00 | 0.01 |  |
| INTERFERON GAMMA RESPONSE | | Hallmark | | 181 | | 0.55 | | 1.49 | 0.00 | 0.02 |  |
| REACTIVE OXIGEN SPECIES PATHWAY | | Hallmark | | 43 | | 0.63 | | 1.45 | 0.02 | 0.03 |  |
| INFLAMMATORY RESPONSE | | Hallmark | | 190 | | 0.53 | | 1.43 | 0.00 | 0.03 |  |
| TNFA SIGNALING VIA NFKB | | Hallmark | | 191 | | 0.52 | | 1.41 | 0.00 | 0.04 |  |
| ALLOGRAFT REJECTION | | Hallmark | | 183 | | 0.52 | | 1.40 | 0.01 | 0.05 |  |
| UNFOLDED PROTEIN RESPONSE | | Hallmark | | 102 | | 0.53 | | 1.37 | 0.03 | 0.06 |  |
| APOPTOSIS | | Hallmark | | 147 | | 0.49 | | 1.32 | 0.03 | 0.11 |  |
| XENOBIOTIC METABOLISM | | Hallmark | | 190 | | 0.48 | | 1.31 | 0.03 | 0.11 |  |
| DNA REPAIR | | Hallmark | | 131 | | 0.50 | | 1.31 | 0.04 | 0.11 |  |
| P53 PATHWAY | | Hallmark | | 189 | | 0.47 | | 1.28 | 0.04 | 0.12 |  |
| LYSOSOME | | CP2:KEGG | | 108 | | 0.78 | | 2.01 | 0.00 | 0.00 |  |
| OXIDATIVE PHOSPHORYLATION | | CP2:KEGG | | 96 | | 0.78 | | 2.00 | 0.00 | 0.00 |  |
| TCA CYCLE AND RESPIRATORY ELECTRON TRANSPORT | | CP2:REACTOME | | 96 | | 0.76 | | 1.96 | 0.00 | 0.00 |  |
| RESPIRATORY ELECTRON TRANSPORT ATP SYNTHESIS BY CHEMIOSMOTIC COUPLING AND HEAT PRODUCTION BY UNCOUPLING PROTEINS | | CP2:REACTOME | | 63 | | 0.80 | | 1.95 | 0.00 | 0.00 |  |
| RESPIRATORY ELECTRON TRANSPORT | | CP2:REACTOME | | 49 | | 0.81 | | 1.92 | 0.00 | 0.00 |  |
| HUNTINGTONS DISEASE | | CP2:KEGG | | 146 | | 0.69 | | 1.85 | 0.00 | 0.00 |  |
| INNATE IMMUNE SYSTEM | | CP2:REACTOME | | 217 | | 0.67 | | 1.83 | 0.00 | 0.00 |  |
| SPHINGOLIMETABOLISM | | CP2:REACTOME | | 57 | | 0.77 | | 1.83 | 0.00 | 0.00 |  |
| TOLL RECEPTOR CASCADES | | CP2:REACTOME | | 105 | | 0.70 | | 1.82 | 0.00 | 0.00 |  |
| GLYCOSPHINGOLIMETABOLISM | | CP2:REACTOME | | 31 | | 0.82 | | 1.81 | 0.00 | 0.00 |  |
| PARKINSONS DISEASE | | CP2:KEGG | | 95 | | 0.71 | | 1.81 | 0.00 | 0.00 |  |
| ALZHEIMERS DISEASE | | CP2:KEGG | | 137 | | 0.68 | | 1.81 | 0.00 | 0.00 |  |
| LEISHMANIA INFECTION | | CP2:KEGG | | 61 | | 0.73 | | 1.77 | 0.00 | 0.00 |  |
| CITRATE CYCLE TCA CYCLE | | CP2:KEGG | | 27 | | 0.81 | | 1.74 | 0.00 | 0.01 |  |
| AMINO SUGAR AND NUCLEOTIDE SUGAR METABOLISM | | CP2:KEGG | | 39 | | 0.76 | | 1.74 | 0.00 | 0.01 |  |
| TOLL LIKE RECEPTOR SIGNALING PATHWAY | | CP2:KEGG | | 89 | | 0.68 | | 1.74 | 0.00 | 0.01 |  |
| FATTY ACID METABOLISM | | CP2:KEGG | | 39 | | 0.76 | | 1.73 | 0.00 | 0.01 |  |
| PROTEASOME PATHWAY | | CP2:BIOCARTA | | 24 | | 0.81 | | 1.72 | 0.00 | 0.01 |  |
| LATENT INFECTION OF HOMO SAPIENS WITH MYCOBACTERIUM TUBERCULOSIS | | CP2:REACTOME | | 28 | | 0.79 | | 1.71 | 0.00 | 0.01 |  |
| MEMBRANE TRAFFICKING | | CP2:REACTOME | | 113 | | 0.65 | | 1.71 | 0.00 | 0.01 |  |
| LYSOSOME | | CP2:KEGG | | 108 | | 0.78 | | 2.01 | 0.00 | 0.00 |  |
| CGGAARNGGCNG_UNKNOWN | | C3-TFT | | 46 | | 0.71 | | 1.67 | 0.00 | 0.03 |  |
| GABP_B | | C3-TFT | | 223 | | 0.59 | | 1.63 | 0.00 | 0.03 |  |
| SREBP1_01 | | C3-TFT | | 147 | | 0.59 | | 1.57 | 0.00 | 0.04 |  |
| ELF1_Q6 | | C3-TFT | | 208 | | 0.58 | | 1.58 | 0.00 | 0.05 |  |
| PU1_Q6 | | C3-TFT | | 202 | | 0.58 | | 1.58 | 0.00 | 0.06 |  |
| TEL2_Q6 | | C3-TFT | | 207 | | 0.55 | | 1.50 | 0.00 | 0.10 |  |
| NRF2_01 | | C3-TFT | | 227 | | 0.54 | | 1.50 | 0.00 | 0.11 |  |
| GGAANCGGAANY_UNKNOWN | | C3-TFT | | 95 | | 0.59 | | 1.50 | 0.01 | 0.12 |  |
| ARNT_02 | | C3-TFT | | 212 | | 0.52 | | 1.43 | 0.00 | 0.23 |  |
| **Gene set enriched in the Rest (HSC & MEP)** | | **Source of ref.** | | **Size** | | **ES** | | **NES** | **NOM p-val** | **FDR**  **q-val** |  |
| HEME METABOLISM | | Hallmark | | 189 | | -0.83 | | -2.80 | 0.00 | 0.00 |  |
| INTERACTION BETWEEN L1 AND ANKYRINS | | CP2:REACTOME | | 21 | | -0.83 | | -1.95 | 0.00 | 0.01 |  |
| GENERIC TRANSCRIPTION PATHWAY | | CP2:REACTOME | | 293 | | -0.55 | | -1.92 | 0.00 | 0.01 |  |
| GATA_Q6 | | C3-TFT | | 175 | | -0.56 | | -1.87 | 0.00 | 0.00 |  |
| GATAAGR_GATA_C | | C3-TFT | | 263 | | -0.52 | | -1.79 | 0.00 | 0.01 |  |
| GATA1_04 | | C3-TFT | | 213 | | -0.48 | | -1.62 | 0.00 | 0.05 |  |
| GATA1_05 | | C3-TFT | | 249 | | -0.47 | | -1.64 | 0.00 | 0.06 |  |
| GATA_C | | C3-TFT | | 233 | | -0.49 | | -1.66 | 0.00 | 0.06 |  |
| STAT3_01 | | C3-TFT | | 19 | | -0.70 | | -1.59 | 0.02 | 0.07 |  |
| LMO2COM_02 | | C3-TFT | | 215 | | -0.44 | | -1.51 | 0.00 | 0.10 |  |
| PAX5_01 | | C3-TFT | | 135 | | -0.48 | | -1.53 | 0.00 | 0.10 |  |
| ACCTGTTG_UNKNOWN | | C3-TFT | | 138 | | -0.47 | | -1.52 | 0.00 | 0.10 |  |
| TTANWNANTGGM_UNKNOWN | | C3-TFT | | 53 | | -0.53 | | -1.48 | 0.02 | 0.12 |  |
| SYATTGTG_UNKNOWN | | C3-TFT | | 203 | | -0.43 | | -1.47 | 0.00 | 0.12 |  |
| MZF1_02 | | C3-TFT | | 211 | | -0.43 | | -1.45 | 0.00 | 0.14 |  |
| GATA1_03 | | C3-TFT | | 224 | | -0.41 | | -1.39 | 0.00 | 0.20 |  |
| **C) GSEA conducted for MEP vs. Rest** | | | | | | | | | | | |
| **Gene sets enriched in the MEP subtype** | | **Source of ref.** | | **Size** | | **ES** | | **NES** | **NOM p-val** | **FDR**  **q-val** | |
| HEME METABOLISM | | Hallmark | | 189 | | 0.92 | | 2.85 | 0.00 | 0.00 | |
| E2F TARGETS | | Hallmark | | 185 | | 0.74 | | 2.27 | 0.00 | 0.00 | |
| G2M CHECKPOINT | | Hallmark | | 186 | | 0.73 | | 2.26 | 0.00 | 0.00 | |
| MYC TARGETS V1 | | Hallmark | | 170 | | 0.62 | | 1.90 | 0.00 | 0.00 | |
| MYC TARGETS V2 | | Hallmark | | 55 | | 0.68 | | 1.79 | 0.00 | 0.00 | |
| SPERMATOGENESIS | | Hallmark | | 128 | | 0.49 | | 1.46 | 0.02 | 0.06 | |
| UV RESPONSE UP | | Hallmark | | 151 | | 0.48 | | 1.46 | 0.01 | 0.05 | |
| UNFOLDED PROTEIN RESPONSE | | Hallmark | | 102 | | 0.49 | | 1.41 | 0.04 | 0.08 | |
| MITOTIC SPINDLE | | Hallmark | | 195 | | 0.43 | | 1.35 | 0.03 | 0.11 | |
| BILE ACID METABOLISM | | Hallmark | | 107 | | 0.46 | | 1.35 | 0.05 | 0.10 | |
| DNA REPAIR | | Hallmark | | 131 | | 0.45 | | 1.34 | 0.04 | 0.10 | |
| PORPHYRIN AND CHLOROPHYLL METABOLISM | | CP2:KEGG | | 27 | | 0.90 | | 2.09 | 0.00 | 0.00 | |
| CELL CYCLE MITOTIC | | CP2:REACTOME | | 273 | | 0.64 | | 2.06 | 0.00 | 0.00 | |
| CELL CYCLE | | CP2:REACTOME | | 348 | | 0.61 | | 2.00 | 0.00 | 0.00 | |
| DNA REPLICATION | | CP2:REACTOME | | 164 | | 0.65 | | 1.99 | 0.00 | 0.00 | |
| MITOTIC M M G1 PHASES | | CP2:REACTOME | | 147 | | 0.66 | | 1.97 | 0.00 | 0.00 | |
| G1 S TRANSITION | | CP2:REACTOME | | 91 | | 0.68 | | 1.93 | 0.00 | 0.00 | |
| G2 M CHECKPOINTS | | CP2:REACTOME | | 40 | | 0.78 | | 1.93 | 0.00 | 0.00 | |
| GLYCINE SERINE AND THREONINE METABOLISM | | CP2:KEGG | | 30 | | 0.80 | | 1.93 | 0.00 | 0.00 | |
| MITOTIC G1 G1 S PHASES | | CP2:REACTOME | | 114 | | 0.66 | | 1.93 | 0.00 | 0.00 | |
| MITOTIC G2 G2 M PHASES | | CP2:REACTOME | | 73 | | 0.70 | | 1.92 | 0.00 | 0.00 | |
| INTERACTION BETWEEN L1 AND ANKYRINS | | CP2:REACTOME | | 21 | | 0.87 | | 1.91 | 0.00 | 0.00 | |
| M G1 TRANSITION | | CP2:REACTOME | | 68 | | 0.69 | | 1.87 | 0.00 | 0.01 | |
| G1 S SPECIFIC TRANSCRIPTION | | CP2:REACTOME | | 15 | | 0.90 | | 1.87 | 0.00 | 0.01 | |
| ACTIVATION OF ATR IN RESPONSE TO REPLICATION STRESS | | CP2:REACTOME | | 35 | | 0.77 | | 1.87 | 0.00 | 0.01 | |
| ACTIVATION OF THE PRE REPLICATIVE COMPLEX | | CP2:REACTOME | | 29 | | 0.79 | | 1.86 | 0.00 | 0.01 | |
| S PHASE | | CP2:REACTOME | | 92 | | 0.65 | | 1.85 | 0.00 | 0.01 | |
| E2F MEDIATED REGULATION OF DNA REPLICATION | | CP2:REACTOME | | 30 | | 0.78 | | 1.84 | 0.00 | 0.01 | |
| CELL CYCLE | | CP2:KEGG | | 117 | | 0.62 | | 1.82 | 0.00 | 0.01 | |
| SYNTHESIS OF DNA | | CP2:REACTOME | | 78 | | 0.64 | | 1.81 | 0.00 | 0.01 | |
| RECRUITMENT OF MITOTIC CENTROSOME PROTEINS AND COMPLEXES | | CP2:REACTOME | | 59 | | 0.68 | | 1.80 | 0.00 | 0.02 | |
| PORPHYRIN AND CHLOROPHYLL METABOLISM | | CP2:KEGG | | 27 | | 0.90 | | 2.09 | 0.00 | 0.00 | |
| GATAAGR_GATA_C | | C3-TFT | | 263 | | 0.61 | | 1.96 | 0.00 | 0.00 | |
| GATA_Q6 | | C3-TFT | | 175 | | 0.61 | | 1.88 | 0.00 | 0.00 | |
| GATA_C | | C3-TFT | | 233 | | 0.55 | | 1.74 | 0.00 | 0.03 | |
| E2F1DP1_01 | | C3-TFT | | 208 | | 0.51 | | 1.60 | 0.00 | 0.03 | |
| E2F_Q4 | | C3-TFT | | 208 | | 0.53 | | 1.68 | 0.00 | 0.04 | |
| E2F1DP2_01 | | C3-TFT | | 208 | | 0.51 | | 1.59 | 0.00 | 0.04 | |
| E2F1_Q4_01 | | C3-TFT | | 201 | | 0.51 | | 1.59 | 0.00 | 0.04 | |
| MYCMAX_01 | | C3-TFT | | 218 | | 0.50 | | 1.58 | 0.00 | 0.04 | |
| E2F_02 | | C3-TFT | | 208 | | 0.51 | | 1.60 | 0.00 | 0.04 | |
| E2F1_Q6 | | C3-TFT | | 210 | | 0.53 | | 1.66 | 0.00 | 0.04 | |
| E2F1_Q6_01 | | C3-TFT | | 213 | | 0.51 | | 1.61 | 0.00 | 0.04 | |
| E2F4DP2_01 | | C3-TFT | | 208 | | 0.51 | | 1.60 | 0.00 | 0.04 | |
| SGCGSSAAA_E2F1DP2_01 | | C3-TFT | | 149 | | 0.54 | | 1.65 | 0.00 | 0.04 | |
| LMO2COM_02 | | C3-TFT | | 215 | | 0.50 | | 1.57 | 0.00 | 0.04 | |
| GATA1_04 | | C3-TFT | | 213 | | 0.51 | | 1.61 | 0.00 | 0.04 | |
| GATA1_05 | | C3-TFT | | 249 | | 0.49 | | 1.56 | 0.00 | 0.04 | |
| E2F_Q6 | | C3-TFT | | 207 | | 0.54 | | 1.68 | 0.00 | 0.04 | |
| E2F4DP1_01 | | C3-TFT | | 212 | | 0.50 | | 1.56 | 0.00 | 0.04 | |
| E2F_Q3 | | C3-TFT | | 201 | | 0.52 | | 1.61 | 0.00 | 0.04 | |
| E2F_Q3_01 | | C3-TFT | | 206 | | 0.52 | | 1.63 | 0.00 | 0.05 | |
| **Gene set enriched in the Rest (HSC & GMP)** | | **Source of ref.** | | **Size** | | **ES** | | **NES** | **NOM p-val** | **FDR**  **q-val** | |
| TNFA SIGNALING VIA NFKB | | Hallmark | | 191 | | -0.73 | | -2.51 | 0.00 | 0.00 | |
| INTERFERON GAMMA RESPONSE | | Hallmark | | 181 | | -0.63 | | -2.15 | 0.00 | 0.00 | |
| IL6 JAK STAT3 SIGNALING | | Hallmark | | 83 | | -0.69 | | -2.10 | 0.00 | 0.00 | |
| INFLAMMATORY RESPONSE | | Hallmark | | 190 | | -0.59 | | -2.01 | 0.00 | 0.00 | |
| COMPLEMENT | | Hallmark | | 188 | | -0.58 | | -1.96 | 0.00 | 0.00 | |
| ALLOGRAFT REJECTION | | Hallmark | | 183 | | -0.57 | | -1.93 | 0.00 | 0.00 | |
| APOPTOSIS | | Hallmark | | 147 | | -0.48 | | -1.59 | 0.00 | 0.01 | |
| INTERFERON ALPHA RESPONSE | | Hallmark | | 87 | | -0.51 | | -1.57 | 0.00 | 0.01 | |
| P53 PATHWAY | | Hallmark | | 189 | | -0.46 | | -1.56 | 0.00 | 0.01 | |
| IL2 STAT5 SIGNALING | | Hallmark | | 187 | | -0.45 | | -1.53 | 0.00 | 0.02 | |
| CHOLESTEROL HOMEOSTASIS | | Hallmark | | 67 | | -0.51 | | -1.53 | 0.01 | 0.02 | |
| PI3K AKT MTOR SIGNALING | | Hallmark | | 99 | | -0.45 | | -1.39 | 0.03 | 0.06 | |
| TGF BETA SIGNALING | | Hallmark | | 52 | | -0.49 | | -1.38 | 0.05 | 0.06 | |
| KRAS SIGNALING UP | | Hallmark | | 184 | | -0.40 | | -1.36 | 0.01 | 0.07 | |
| LEISHMANIA INFECTION | | CP2:KEGG | | 61 | | -0.80 | | -2.32 | 0.00 | 0.00 | |
| BCR 5PATHWAY | | CP2:PID | | 64 | | -0.76 | | -2.26 | 0.00 | 0.00 | |
| B CELL RECEPTOR SIGNALING PATHWAY | | CP2:KEGG | | 68 | | -0.75 | | -2.21 | 0.00 | 0.00 | |
| FC GAMMA R MEDIATED PHAGOCYTOSIS | | CP2:KEGG | | 81 | | -0.72 | | -2.19 | 0.00 | 0.00 | |
| IL8 CXCR2 PATHWAY | | CP2:PID | | 32 | | -0.86 | | -2.18 | 0.00 | 0.00 | |
| NATURAL KILLER CELL MEDIATED CYTOTOXICITY | | CP2:KEGG | | 106 | | -0.68 | | -2.16 | 0.00 | 0.00 | |
| TOLL RECEPTOR CASCADES | | CP2:REACTOME | | 105 | | -0.68 | | -2.15 | 0.00 | 0.00 | |
| CXCR4 PATHWAY | | CP2:PID | | 97 | | -0.68 | | -2.13 | 0.00 | 0.00 | |
| THROMBIN PAR1 PATHWAY | | CP2:PID | | 42 | | -0.79 | | -2.12 | 0.00 | 0.00 | |
| CHEMOKINE SIGNALING PATHWAY | | CP2:KEGG | | 170 | | -0.63 | | -2.12 | 0.00 | 0.00 | |
| INTERFERON GAMMA SIGNALING | | CP2:REACTOME | | 51 | | -0.75 | | -2.11 | 0.00 | 0.00 | |
| TOLL LIKE RECEPTOR SIGNALING PATHWAY | | CP2:KEGG | | 89 | | -0.68 | | -2.11 | 0.00 | 0.00 | |
| CYTOKINE SIGNALING IN IMMUNE SYSTEM | | CP2:REACTOME | | 237 | | -0.61 | | -2.11 | 0.00 | 0.00 | |
| INNATE IMMUNE SYSTEM | | CP2:REACTOME | | 217 | | -0.61 | | -2.11 | 0.00 | 0.00 | |
| IL8 CXCR1 PATHWAY | | CP2:PID | | 26 | | -0.84 | | -2.10 | 0.00 | 0.00 | |
| PDGFRB PATHWAY | | CP2:PID | | 121 | | -0.65 | | -2.10 | 0.00 | 0.00 | |
| IFNG PATHWAY | | CP2:PID | | 38 | | -0.79 | | -2.09 | 0.00 | 0.00 | |
| FCER1 PATHWAY | | CP2:BIOCARTA | | 37 | | -0.79 | | -2.08 | 0.00 | 0.00 | |
| EPITHELIAL CELL SIGNALING IN HELICOBACTER PYLORI INFECTION | | CP2:KEGG | | 64 | | -0.71 | | -2.08 | 0.00 | 0.00 | |
| FAK PATHWAY | | CP2:PID | | 56 | | -0.72 | | -2.06 | 0.00 | 0.00 | |
| PEA3_Q6 | | C3-TFT | | 233 | | -0.55 | | -1.90 | 0.00 | 0.00 | |
| PU1_Q6 | | C3-TFT | | 202 | | -0.56 | | -1.91 | 0.00 | 0.00 | |
| ETS_Q4 | | C3-TFT | | 215 | | -0.51 | | -1.78 | 0.00 | 0.01 | |
| CREBP1_01 | | C3-TFT | | 159 | | -0.53 | | -1.76 | 0.00 | 0.01 | |
| ELF1_Q6 | | C3-TFT | | 208 | | -0.52 | | -1.79 | 0.00 | 0.01 | |
| TEL2_Q6 | | C3-TFT | | 207 | | -0.51 | | -1.76 | 0.00 | 0.01 | |
| MAF_Q6 | | C3-TFT | | 230 | | -0.50 | | -1.74 | 0.00 | 0.01 | |
| IRF1_Q6 | | C3-TFT | | 225 | | -0.49 | | -1.71 | 0.00 | 0.01 | |
| CGTSACG_PAX3_B | | C3-TFT | | 133 | | -0.52 | | -1.70 | 0.00 | 0.01 | |
| RGAGGAARY_PU1_Q6 | | C3-TFT | | 443 | | -0.45 | | -1.68 | 0.00 | 0.01 | |
| NFKB_Q6_01 | | C3-TFT | | 216 | | -0.47 | | -1.62 | 0.00 | 0.03 | |
| ER_Q6_02 | | C3-TFT | | 226 | | -0.43 | | -1.51 | 0.00 | 0.06 | |
| ICSBP_Q6 | | C3-TFT | | 223 | | -0.44 | | -1.52 | 0.00 | 0.06 | |
| AML_Q6 | | C3-TFT | | 230 | | -0.43 | | -1.52 | 0.00 | 0.06 | |
| AP1_Q4_01 | | C3-TFT | | 239 | | -0.43 | | -1.50 | 0.00 | 0.06 | |
| COREBINDINGFACTOR_Q6 | | C3-TFT | | 230 | | -0.43 | | -1.50 | 0.00 | 0.06 | |
| IRF_Q6 | | C3-TFT | | 215 | | -0.44 | | -1.51 | 0.00 | 0.06 | |
| CCAWWNAAGG_SRF_Q4 | | C3-TFT | | 74 | | -0.51 | | -1.53 | 0.01 | 0.07 | |
| SRF_01 | | C3-TFT | | 48 | | -0.55 | | -1.53 | 0.02 | 0.07 | |
| PPARA_02 | | C3-TFT | | 113 | | -0.49 | | -1.54 | 0.00 | 0.07 | |

GSEA was conducted in the assignment probability filtered clinical meta-cohort (N = 483: N_GSE6891_ = 347, N_TCGA_ = 136), restricting analysis to samples that passed the MAGS assignment probability threshold of ≥ 0.75. Each contrast was investigated using three gene set collections retrieved from the MSigDB: the *Hallmark* collection (50 gene sets), the *C2-CP* collection of canonical pathways (1329 gene sets), and the C3-TFT collection of transcription factor targets (615 gene sets). Gene sets with a normalized p-value ≤ 0.01 and FDR ≤ 0.25 were identified as enriched. Gene sets significantly enriched in the MAGS subtype of interest indicated an upregulation of those gene sets compared to the “Rest-group”, representing the other two subtypes. Vice versa, gene sets significantly enriched in the “Rest-group” indicated a down-regulation of those gene sets in the subtype of interest compared to the other two subtypes. Only significant gene sets were shown in the table and significantly enriched gene sets were limited to the top 20 enriched gene sets per gene set collection. Abbreviations: Size, number of genes included in gene set; ES, Enrichment score; NES, normalized enrichment score; NOM p-val, multiple test corrected p-value for gene set size normalized ES; FDR q-val, false discovery rate of normalized ES; HSC, hematopoietic stem cells; GMP, granulocytic-monocytic progenitors; MEP, megakaryocyte-erythroid progenitors; UC, unclassified
