# Supplementary material for: Normal myeloid progenitor cell subset-associated gene signatures for acute myeloid leukaemia subtyping with prognostic impact
Source: PLoS One. 2020 Apr 23;15(4):e0229593. doi: 10.1371/journal.pone.0229593 (PMC7179860; doi:10.1371/journal.pone.0229593)
Supplement: S8 Table — (DOCX) [file pone.0229593.s009.docx]

**Supplemental Table S8:** MAGS subtype-specific mutation patterns for: **A)** seven AML oncogenes investigated in the mutation filtered clinical meta-cohort (N = 587: N_GSE6891_ = 457, N_TCGA_ = 130), restricted to samples with recorded mutation information; and **B)** 112 selected genes investigated in the mutation filtered TCGA cohort (N = 130), restricted to samples with recorded mutation information from exome-wide sequence data. Two-sided Fisher´s exact tests were conducted to test for association between MAGS subtypes and gene aberrations.

| **A) Meta-cohort (N=587)** | | | | | | | | | | | | | |
| --- | --- | --- | --- | --- | --- | --- | --- | --- | --- | --- | --- | --- | --- |
| **Gene** | **HSC (N=163)** | | | **MEP (N=147)** | | | **GMP (N=191)** | | | **UC (N=86)** | | | **P-Value** |
|  | N_mut_ | %_HSC_ | %_total_ | N_mut_ | %_MEP_ | %_total_ | N_mut_ | %_GMP_ | %_total_ | N_mut_ | %_UC_ | %_total_ |  |
| CEBPA | 3 | 1.8 | 0.5 | 24 | 16.3 | 4.1 | 3 | 1.6 | 0.5 | 5 | 5.8 | 0.9 | **5.79e-08***** |
| IDH2^a^ | 20 | 12.3 | 3.4 | 11 | 7.5 | 1.9 | 7 | 3.7 | 1.2 | 9 | 10.5 | 1.5 | **0.015*** |
| FLT3 itd | 41 | 25.2 | 7.0 | 21 | 14.3 | 3.6 | 43 | 22.5 | 7.3 | 21 | 24.4 | 3.6 | 0.081 |
| FLT3 tkd^b^ | 15 | 9.2 | 2.6 | 13 | 8.8 | 2.2 | 30 | 15.7 | 5.1 | 6 | 7.0 | 1.0 | 0.087 |
| KRAS | 2 | 1.2 | 0.3 | 0 | 0.0 | 0.0 | 6 | 3.1 | 1.0 | 2 | 2.3 | 0.3 | 0.110 |
| NPM1 | 37 | 22.7 | 6.3 | 33 | 22.4 | 5.6 | 61 | 31.9 | 10.4 | 21 | 24.4 | 3.6 | 0.149 |
| NRAS | 13 | 8.0 | 2.2 | 12 | 8.2 | 2.0 | 20 | 10.5 | 3.4 | 8 | 9.3 | 1.4 | 0.851 |
| IDH1^c^ | 11 | 6.7 | 1.9 | 11 | 7.5 | 1.9 | 14 | 7.3 | 2.4 | 4 | 4.7 | 0.7 | 0.876 |
| **B) TCGA cohort (N=130)** | | | | | | | | | | | | | |
| **Gene** | **HSC (N=38)** | | | **MEP (N=29)** | | | **GMP (N=43)** | | | **UC (N=20)** | | | **P-Value** |
|  | N_mut_ | %_HSC_ | %_total_ | N_mut_ | %_GMP_ | %_total_ | N_mut_ | %_MEP_ | %_total_ | N_mut_ | %_UC_ | %_total_ |  |
| RUNX1 | 9 | 23.7 | 6.9 | 0 | 0.0 | 0.0 | 2 | 4.7 | 1.5 | 2 | 10.0 | 1.5 | **0.005**** |
| RUNX1T1 | 0 | 0.0 | 0.0 | 0 | 0.0 | 0.0 | 1 | 2.3 | 0.8 | 3 | 15.0 | 2.3 | **0.014*** |
| TP53 | 5 | 3.8 | 3.8 | 5 | 17.2 | 3.8 | 0 | 0.0 | 0.0 | 2 | 10.0 | 1.5 | **0.02*** |
| WT1 | 0 | 0.0 | 0.0 | 2 | 6.9 | 1.5 | 6 | 14.0 | 4.6 | 0 | 0.0 | 0.0 | **0.031*** |
| DNMT3A | 10 | 7.7 | 7.7 | 4 | 13.8 | 3.1 | 3 | 7.0 | 2.3 | 1 | 5.0 | 0.8 | 0.062 |
| ATXN7L1 | 0 | 0.0 | 0.0 | 2 | 6.9 | 1.5 | 0 | 0.0 | 0.0 | 0 | 0.0 | 0.0 | 0.071 |
| MYC | 0 | 0.0 | 0.0 | 2 | 6.9 | 1.5 | 0 | 0.0 | 0.0 | 0 | 0.0 | 0.0 | 0.071 |
| KIT | 2 | 1.5 | 1.5 | 1 | 3.4 | 0.8 | 1 | 2.3 | 0.8 | 4 | 20.0 | 3.1 | 0.079 |
| IDH1 | 4 | 3.1 | 3.1 | 1 | 3.4 | 0.8 | 0 | 0.0 | 0.0 | 1 | 5.0 | 0.8 | 0.116 |
| NRAS | 0 | 0.0 | 0.0 | 3 | 10.3 | 2.3 | 3 | 7.0 | 2.3 | 2 | 10.0 | 1.5 | 0.148 |
| CTNNA1 | 0 | 0.0 | 0.0 | 0 | 0.0 | 0.0 | 0 | 0.0 | 0.0 | 1 | 5.0 | 0.8 | 0.154 |
| KDM2B | 0 | 0.0 | 0.0 | 0 | 0.0 | 0.0 | 0 | 0.0 | 0.0 | 1 | 5.0 | 0.8 | 0.154 |
| ASXL1 | 3 | 2.3 | 2.3 | 1 | 3.4 | 0.8 | 0 | 0.0 | 0.0 | 1 | 5.0 | 0.8 | 0.223 |
| CREBBP | 0 | 0.0 | 0.0 | 2 | 6.9 | 1.5 | 1 | 2.3 | 0.8 | 0 | 0.0 | 0.0 | 0.284 |
| CEBPA | 0 | 0.0 | 0.0 | 2 | 6.9 | 1.5 | 1 | 2.3 | 0.8 | 1 | 5.0 | 0.8 | 0.305 |
| KDM6A | 1 | 0.8 | 0.8 | 0 | 0.0 | 0.0 | 0 | 0.0 | 0.0 | 1 | 5.0 | 0.8 | 0.315 |
| TET1 | 0 | 0.0 | 0.0 | 1 | 3.4 | 0.8 | 3 | 7.0 | 2.3 | 0 | 0.0 | 0.0 | 0.336 |
| STK17B | 0 | 0.0 | 0.0 | 1 | 3.4 | 0.8 | 0 | 0.0 | 0.0 | 0 | 0.0 | 0.0 | 0.377 |
| ARHGAP26 | 0 | 0.0 | 0.0 | 1 | 3.4 | 0.8 | 0 | 0.0 | 0.0 | 0 | 0.0 | 0.0 | 0.377 |
| JAK2 | 0 | 0.0 | 0.0 | 1 | 3.4 | 0.8 | 0 | 0.0 | 0.0 | 0 | 0.0 | 0.0 | 0.377 |
| KDM5A | 0 | 0.0 | 0.0 | 1 | 3.4 | 0.8 | 0 | 0.0 | 0.0 | 0 | 0.0 | 0.0 | 0.377 |
| GNAS | 0 | 0.0 | 0.0 | 1 | 3.4 | 0.8 | 0 | 0.0 | 0.0 | 0 | 0.0 | 0.0 | 0.377 |
| SF3A1 | 0 | 0.0 | 0.0 | 1 | 3.4 | 0.8 | 0 | 0.0 | 0.0 | 0 | 0.0 | 0.0 | 0.377 |
| JAK3 | 0 | 0.0 | 0.0 | 1 | 3.4 | 0.8 | 0 | 0.0 | 0.0 | 0 | 0.0 | 0.0 | 0.377 |
| NPM1 | 4 | 3.1 | 3.1 | 3 | 10.3 | 2.3 | 6 | 14.0 | 4.6 | 0 | 0.0 | 0.0 | 0.379 |
| ATRX | 0 | 0.0 | 0.0 | 0 | 0.0 | 0.0 | 1 | 2.3 | 0.8 | 1 | 5.0 | 0.8 | 0.417 |
| ZEB2 | 0 | 0.0 | 0.0 | 0 | 0.0 | 0.0 | 2 | 4.7 | 1.5 | 0 | 0.0 | 0.0 | 0.525 |
| SF3B1 | 0 | 0.0 | 0.0 | 0 | 0.0 | 0.0 | 2 | 4.7 | 1.5 | 0 | 0.0 | 0.0 | 0.525 |
| IDH2 | 5 | 3.8 | 3.8 | 1 | 3.4 | 0.8 | 3 | 7.0 | 2.3 | 1 | 5.0 | 0.8 | 0.554 |
| FLT3 | 7 | 5.4 | 5.4 | 2 | 6.9 | 1.5 | 5 | 11.6 | 3.8 | 2 | 10.0 | 1.5 | 0.583 |
| KRAS | 2 | 1.5 | 1.5 | 0 | 0.0 | 0.0 | 3 | 7.0 | 2.3 | 1 | 5.0 | 0.8 | 0.583 |
| RAD50 | 0 | 0.0 | 0.0 | 1 | 3.4 | 0.8 | 2 | 4.7 | 1.5 | 0 | 0.0 | 0.0 | 0.596 |
| RAD21 | 0 | 0.0 | 0.0 | 1 | 3.4 | 0.8 | 2 | 4.7 | 1.5 | 0 | 0.0 | 0.0 | 0.596 |
| GATA2 | 0 | 0.0 | 0.0 | 1 | 3.4 | 0.8 | 2 | 4.7 | 1.5 | 0 | 0.0 | 0.0 | 0.596 |
| MMD2 | 1 | 0.8 | 0.8 | 1 | 3.4 | 0.8 | 0 | 0.0 | 0.0 | 0 | 0.0 | 0.0 | 0.656 |
| U2AF2 | 1 | 0.8 | 0.8 | 0 | 0.0 | 0.0 | 0 | 0.0 | 0.0 | 0 | 0.0 | 0.0 | 0.669 |
| MN1 | 1 | 0.8 | 0.8 | 0 | 0.0 | 0.0 | 0 | 0.0 | 0.0 | 0 | 0.0 | 0.0 | 0.669 |
| EGFR | 1 | 0.8 | 0.8 | 0 | 0.0 | 0.0 | 0 | 0.0 | 0.0 | 0 | 0.0 | 0.0 | 0.669 |
| ETV6 | 1 | 0.8 | 0.8 | 0 | 0.0 | 0.0 | 0 | 0.0 | 0.0 | 0 | 0.0 | 0.0 | 0.669 |
| SH2B3 | 1 | 0.8 | 0.8 | 0 | 0.0 | 0.0 | 0 | 0.0 | 0.0 | 0 | 0.0 | 0.0 | 0.669 |
| TCF4 | 1 | 0.8 | 0.8 | 0 | 0.0 | 0.0 | 0 | 0.0 | 0.0 | 0 | 0.0 | 0.0 | 0.669 |
| SRSF2 | 1 | 0.8 | 0.8 | 0 | 0.0 | 0.0 | 0 | 0.0 | 0.0 | 0 | 0.0 | 0.0 | 0.669 |
| PHF6 | 2 | 1.5 | 1.5 | 0 | 0.0 | 0.0 | 1 | 2.3 | 0.8 | 0 | 0.0 | 0.0 | 0.68 |
| TET2 | 1 | 0.8 | 0.8 | 0 | 0.0 | 0.0 | 1 | 2.3 | 0.8 | 1 | 5.0 | 0.8 | 0.772 |
| ABCA12 | 0 | 0.0 | 0.0 | 1 | 3.4 | 0.8 | 1 | 2.3 | 0.8 | 0 | 0.0 | 0.0 | 0.805 |
| NSD1 | 0 | 0.0 | 0.0 | 1 | 3.4 | 0.8 | 1 | 2.3 | 0.8 | 0 | 0.0 | 0.0 | 0.805 |
| MET | 0 | 0.0 | 0.0 | 1 | 3.4 | 0.8 | 1 | 2.3 | 0.8 | 0 | 0.0 | 0.0 | 0.805 |
| NUP98 | 0 | 0.0 | 0.0 | 1 | 3.4 | 0.8 | 1 | 2.3 | 0.8 | 0 | 0.0 | 0.0 | 0.805 |
| EP300 | 0 | 0.0 | 0.0 | 1 | 3.4 | 0.8 | 1 | 2.3 | 0.8 | 0 | 0.0 | 0.0 | 0.805 |
| EZH2 | 0 | 0.0 | 0.0 | 1 | 3.4 | 0.8 | 1 | 2.3 | 0.8 | 0 | 0.0 | 0.0 | 0.805 |
| NLRP1 | 0 | 0.0 | 0.0 | 1 | 3.4 | 0.8 | 1 | 2.3 | 0.8 | 0 | 0.0 | 0.0 | 0.805 |
| STAG2 | 0 | 0.0 | 0.0 | 1 | 3.4 | 0.8 | 1 | 2.3 | 0.8 | 0 | 0.0 | 0.0 | 0.805 |
| NF1 | 1 | 0.8 | 0.8 | 1 | 3.4 | 0.8 | 2 | 4.7 | 1.5 | 1 | 5.0 | 0.8 | 1 |
| PHF12 | 1 | 0.8 | 0.8 | 0 | 0.0 | 0.0 | 1 | 2.3 | 0.8 | 0 | 0.0 | 0.0 | 1 |
| RORC | 0 | 0.0 | 0.0 | 0 | 0.0 | 0.0 | 1 | 2.3 | 0.8 | 0 | 0.0 | 0.0 | 1 |
| CDH1 | 0 | 0.0 | 0.0 | 0 | 0.0 | 0.0 | 1 | 2.3 | 0.8 | 0 | 0.0 | 0.0 | 1 |
| ACTR5 | 0 | 0.0 | 0.0 | 0 | 0.0 | 0.0 | 1 | 2.3 | 0.8 | 0 | 0.0 | 0.0 | 1 |
| PDGFRA | 0 | 0.0 | 0.0 | 0 | 0.0 | 0.0 | 1 | 2.3 | 0.8 | 0 | 0.0 | 0.0 | 1 |
| SRPK2 | 0 | 0.0 | 0.0 | 0 | 0.0 | 0.0 | 1 | 2.3 | 0.8 | 0 | 0.0 | 0.0 | 1 |
| RINT1 | 0 | 0.0 | 0.0 | 0 | 0.0 | 0.0 | 1 | 2.3 | 0.8 | 0 | 0.0 | 0.0 | 1 |
| SF1 | 0 | 0.0 | 0.0 | 0 | 0.0 | 0.0 | 1 | 2.3 | 0.8 | 0 | 0.0 | 0.0 | 1 |
| ELF1 | 0 | 0.0 | 0.0 | 0 | 0.0 | 0.0 | 1 | 2.3 | 0.8 | 0 | 0.0 | 0.0 | 1 |
| MECOM | 0 | 0.0 | 0.0 | 0 | 0.0 | 0.0 | 1 | 2.3 | 0.8 | 0 | 0.0 | 0.0 | 1 |
| DNMT1 | 0 | 0.0 | 0.0 | 0 | 0.0 | 0.0 | 1 | 2.3 | 0.8 | 0 | 0.0 | 0.0 | 1 |
| CBLC | 0 | 0.0 | 0.0 | 0 | 0.0 | 0.0 | 1 | 2.3 | 0.8 | 0 | 0.0 | 0.0 | 1 |
| PTPN11 | 1 | 0.8 | 0.8 | 1 | 3.4 | 0.8 | 1 | 2.3 | 0.8 | 0 | 0.0 | 0.0 | 1 |
| CBL | 0 | 0.0 | 0.0 | 0 | 0.0 | 0.0 | 1 | 2.3 | 0.8 | 0 | 0.0 | 0.0 | 1 |
| BCOR | 0 | 0.0 | 0.0 | 0 | 0.0 | 0.0 | 1 | 2.3 | 0.8 | 0 | 0.0 | 0.0 | 1 |

Significance levels: * ≤ 0.05, ** ≤ 0.01, *** ≤ 0.001; Abbreviations: HSC, hematopoietic stem cells; GMP, granulocytic-monocytic progenitors; MEP, megakaryocyte-erythroid progenitors; UC, unclassified; *FLT3* itd, in-frame insertions in the JM domain of the *FLT3* gene (position aa572 to aa610); *FLT3* tkd, point mutations in the second tyrosine kinase domain (position 738-958) of the *FLT3* gene; ^a^ missing mutation records in *IDH2* (N_NA_ = 3, HSC: 1, GMP: 1, MEP: 0, UC: 1); ^b^ missing mutation records in *FLT3* tkd (N_NA_ = 1, HSC: 0, GMP: 1, MEP: 0, UC: 0); ^c^ missing mutation records in *IDH2* (N_NA_ = 3, HSC: 1, GMP: 1, MEP: 0, UC: 1)
