# Supplementary material for: Normal myeloid progenitor cell subset-associated gene signatures for acute myeloid leukaemia subtyping with prognostic impact
Source: PLoS One. 2020 Apr 23;15(4):e0229593. doi: 10.1371/journal.pone.0229593 (PMC7179860; doi:10.1371/journal.pone.0229593)
Supplement: S9 Table — (DOCX) [file pone.0229593.s010.docx]

**Supplemental Table S9:** Association between the MAGS and FAB classification. Associations were investigated in the molecular subtype filtered meta-cohort^a^, including samples with recorded FAB classification and limited to FAB subtypes M0-M7, and unknown (N = 621, N_GSE6891_ = 439, N_TCGA_ = 182).

| **FAB\MAGS** | **HSC (%)** | **MEP (%)** | **GMP (%)** | **UC (%)** | **Total** | **P-Value** |
| --- | --- | --- | --- | --- | --- | --- |
| M0 | 22 (70.97) | 4 (12.90) | 3 (9.68) | 2 (6.45) | 31 | **3.70e-06^***^** |
| M1 | 52 (38.24) | 37 (27.21) | 24 (17.65) | 23 (16.91) | 136 | **8.00e-05^***^** |
| M2 | 46 (31.51) | 43 (29.45) | 27 (18.49) | 30 (20.55) | 146 | **1.80e-04^**^** |
| M3 | 1 (2.44) | 27 (65.85) | 11 (26.83) | 2 (4.88) | 41 | **6.16e-09^***^** |
| M4 | 20 (17.39) | 19 (16.52) | 52 (45.22) | 24 (20.87) | 115 | **1.95e-04^**^** |
| M4eo | 1 (20.00) | 0 (0.00) | 3 (60.00) | 1 (20.00) | 5 | 0.49 |
| M5 | 23 (18.40) | 11 (8.80) | 82 (65.60) | 9 (7.20) | 125 | **2.10e-16^***^** |
| M6 | 1 (11.11) | 8 (88.89) | 0 (0.00) | 0 (0.00) | 9 | **1.88e-04^***^** |
| M7 | 0 (0.00) | 3 (100.00) | 0 (0.00) | 0 (0.00) | 3 | **0.02^*^** |
| unknown | 4 (40.00) | 4 (40.00) | 1 (10.00) | 1 (10.00) | 10 | 0.31 |
| total | 170 | 156 | 203 | 92 | 621 |  |

Subtype assignment frequencies were calculated within each FAB subtype. Tests for significant association between MAGS and FAB subtypes were conducted using two-sided Fisher´s exact tests. Significance levels: * ≤ 0.05, ** ≤ 0.01, *** ≤ 0.001; Abbreviations: HSC, hematopoietic stem cells; MEP, megakaryocyte-erythroid progenitors; GMP, granulocytic-monocytic progenitors; UC, unclassified; ^a^ Samples of FAB subtype Mx (N = 1), RAEB (N = 4), and RAEB-t (N = 13) were removed from this analysis.
