# Supplementary material for: Normal myeloid progenitor cell subset-associated gene signatures for acute myeloid leukaemia subtyping with prognostic impact
Source: PLoS One. 2020 Apr 23;15(4):e0229593. doi: 10.1371/journal.pone.0229593 (PMC7179860; doi:10.1371/journal.pone.0229593)

**Supplemental Figure S1:** Identification of regularization-parameters through cross validation in the training-cohort (N = 18).


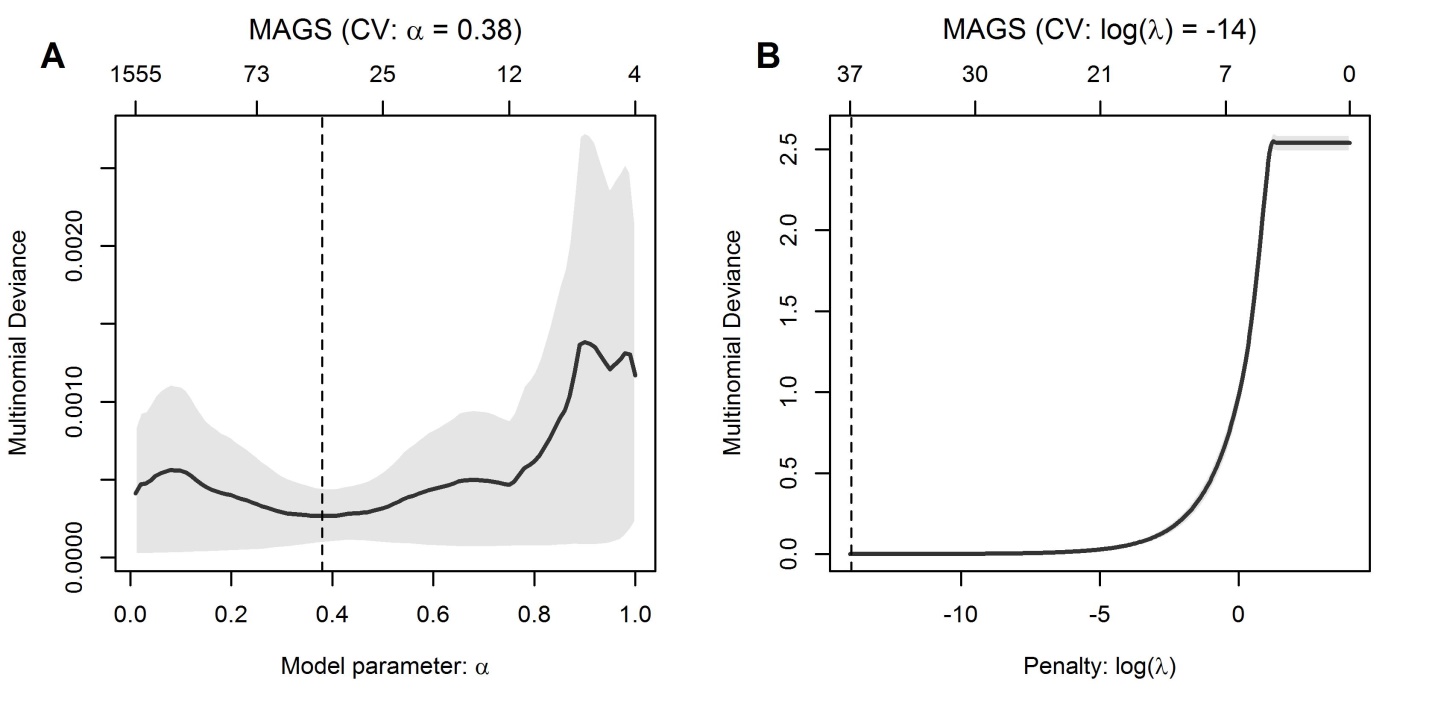

Supplement: S1 Fig — (DOCX) [file pone.0229593.s011.docx]
