# Supplementary material for: Normal myeloid progenitor cell subset-associated gene signatures for acute myeloid leukaemia subtyping with prognostic impact
Source: PLoS One. 2020 Apr 23;15(4):e0229593. doi: 10.1371/journal.pone.0229593 (PMC7179860; doi:10.1371/journal.pone.0229593)

**Supplemental Figure S2:** Venn Diagram of MAGS subtype-specific differentially expressed genes conducted for three contrasts (HSC vs. Rest, GMP vs. Rest and MEP vs. Rest).


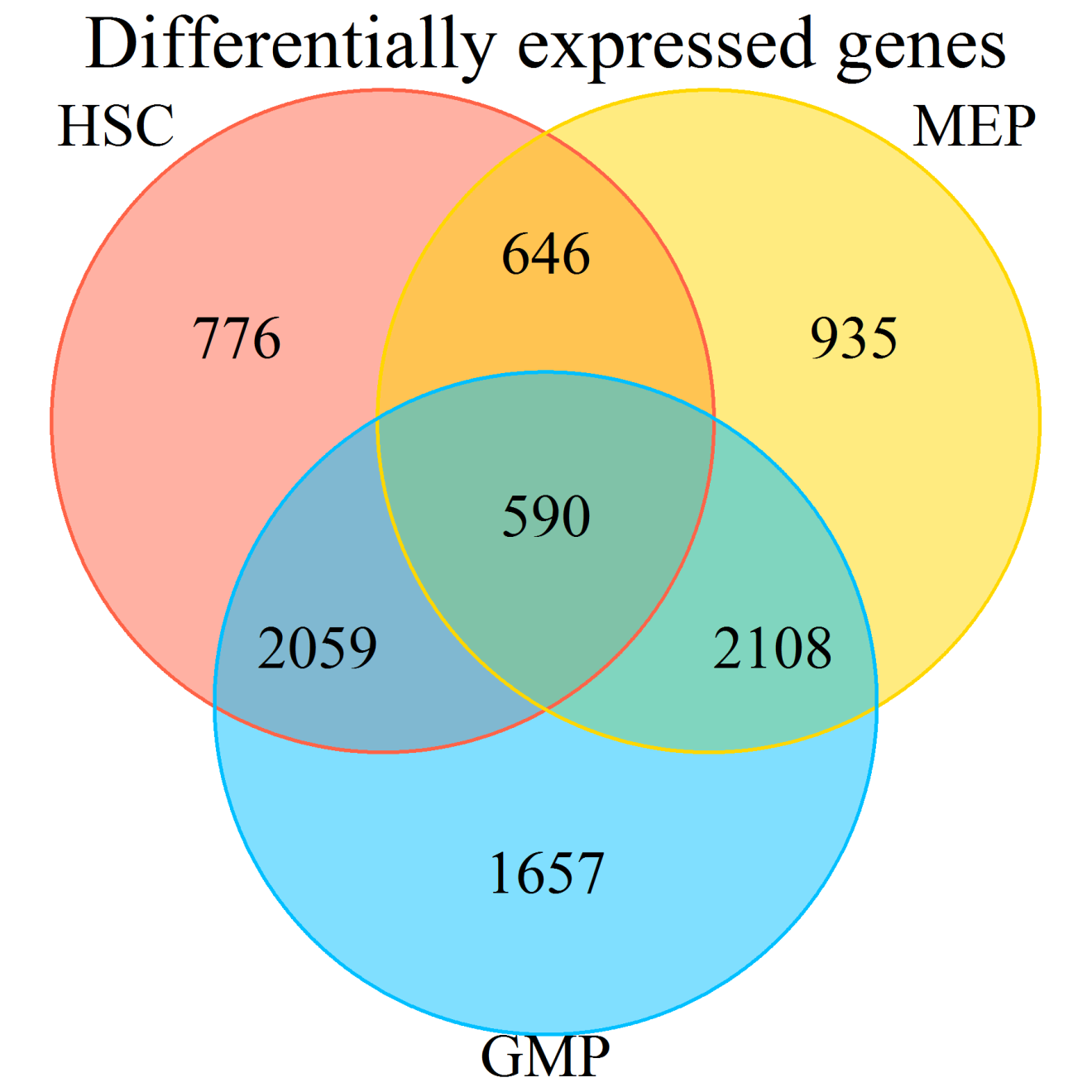

Supplement: S2 Fig — (DOCX) [file pone.0229593.s012.docx]
