# Supplementary material for: Normal myeloid progenitor cell subset-associated gene signatures for acute myeloid leukaemia subtyping with prognostic impact
Source: PLoS One. 2020 Apr 23;15(4):e0229593. doi: 10.1371/journal.pone.0229593 (PMC7179860; doi:10.1371/journal.pone.0229593)
Supplement: S3 Fig — (PDF) [file pone.0229593.s013.pdf]

Supplemental Figure S3: GSEA enrichment plots for **A)** the HSC low cell-cycle activity profile; **B)** the GMP high metabolism profile; **C)** the GMP innate immune activation profile; **D)** the MEP high cell-cycle activity profile; and **E)** the MEP impaired innate immune activity profile. Only top ranking significantly enriched gene sets are presented.

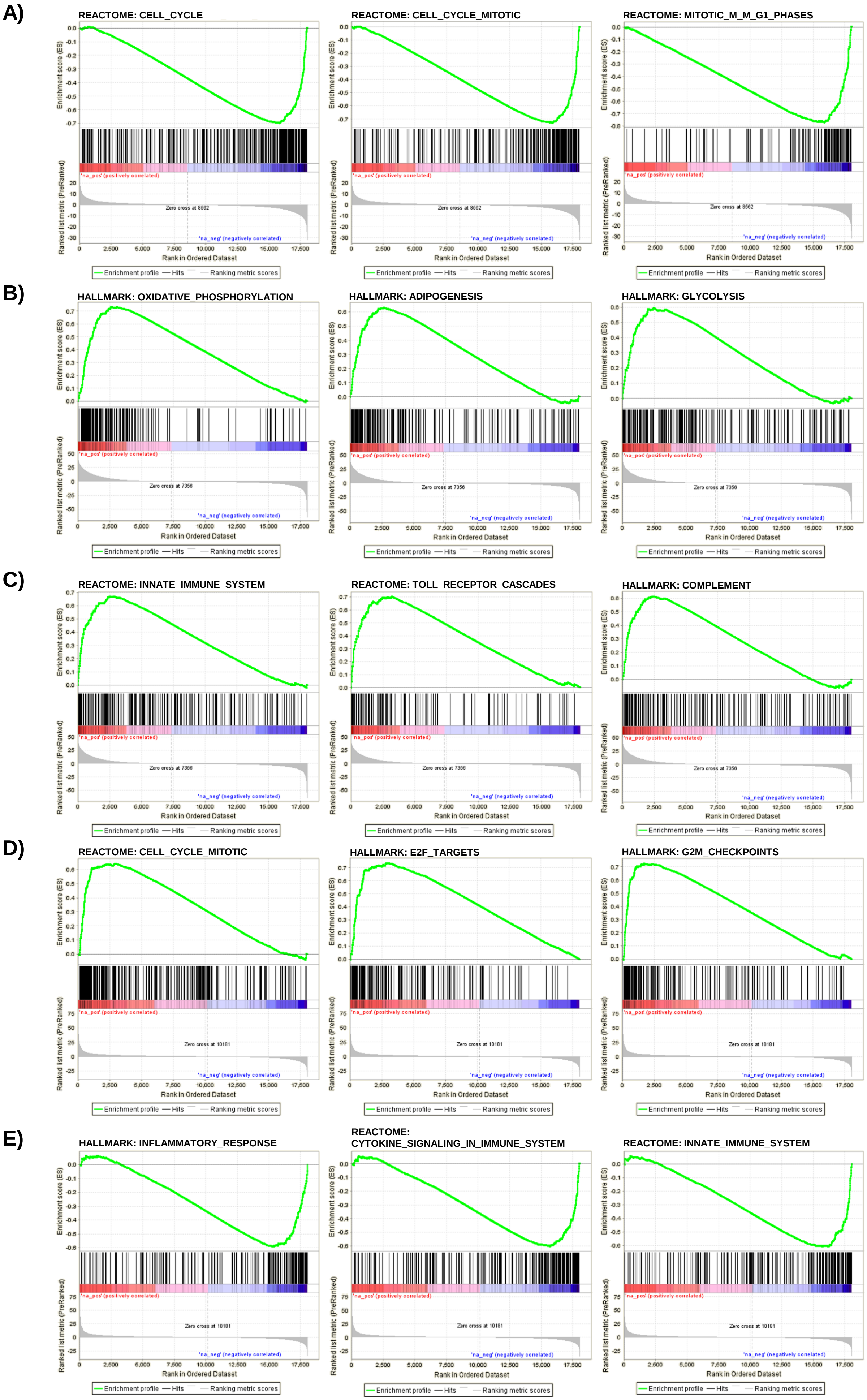
A)
REACTOME: CELL_CYCLE
REACTOME: CELL_CYCLE_MITOTIC
REACTOME: MITOTIC_M_M_G1_PHASES
B)
HALLMARK: OXIDATIVE_PHOSPHORYLATION
HALLMARK: ADIPOGENESIS
HALLMARK: GLYCOLYSIS
C)
REACTOME: INNATE_IMMUNE_SYSTEM
REACTOME: TOLL_RECEPTOR_CASCADES
HALLMARK: COMPLEMENT
D)
REACTOME: CELL_CYCLE_MITOTIC
HALLMARK: E2F_TARGETS
HALLMARK: G2M_CHECKPOINTS
E)
HALLMARK: INFLAMMATORY_RESPONSE
REACTOME: CYTOKINE_SIGNALING_IN_IMMUNE_SYSTEM
REACTOME: INNATE_IMMUNE_SYSTEM
Enrichment score (ES)
Ranked list metric (PreRanked)
Rank in Ordered Dataset
'na_pos' (positively correlated)
'na_neg' (negatively correlated)
Zero cross at 8562
Zero cross at 7356
Zero cross at 10181
Enrichment profile
Hits
Ranking metric scores
