# Supplementary material for: Normal myeloid progenitor cell subset-associated gene signatures for acute myeloid leukaemia subtyping with prognostic impact
Source: PLoS One. 2020 Apr 23;15(4):e0229593. doi: 10.1371/journal.pone.0229593 (PMC7179860; doi:10.1371/journal.pone.0229593)

**Supplemental Figure S4:** Prognostic validation of assigned MAGS subtypes for the clinical meta-cohort (N = 691) using three different frequency cut-offs for assignment of unclassified samples: **A)** 90% MAGS subtype assigned and 10% assigned as unclassified; **B)** 80% of samples MAGS subtype assigned and 20% assigned as unclassified; and **C)** 75% of samples MAGS subtype assigned and 25% assigned as unclassified. Kaplan Meier survival curves were generated for overall survival and p-values were estimated using a log-rank test. Number at risk per MAGS subtype is provided for each frequency cut-off. Only samples with complete survival information were included in the analysis.


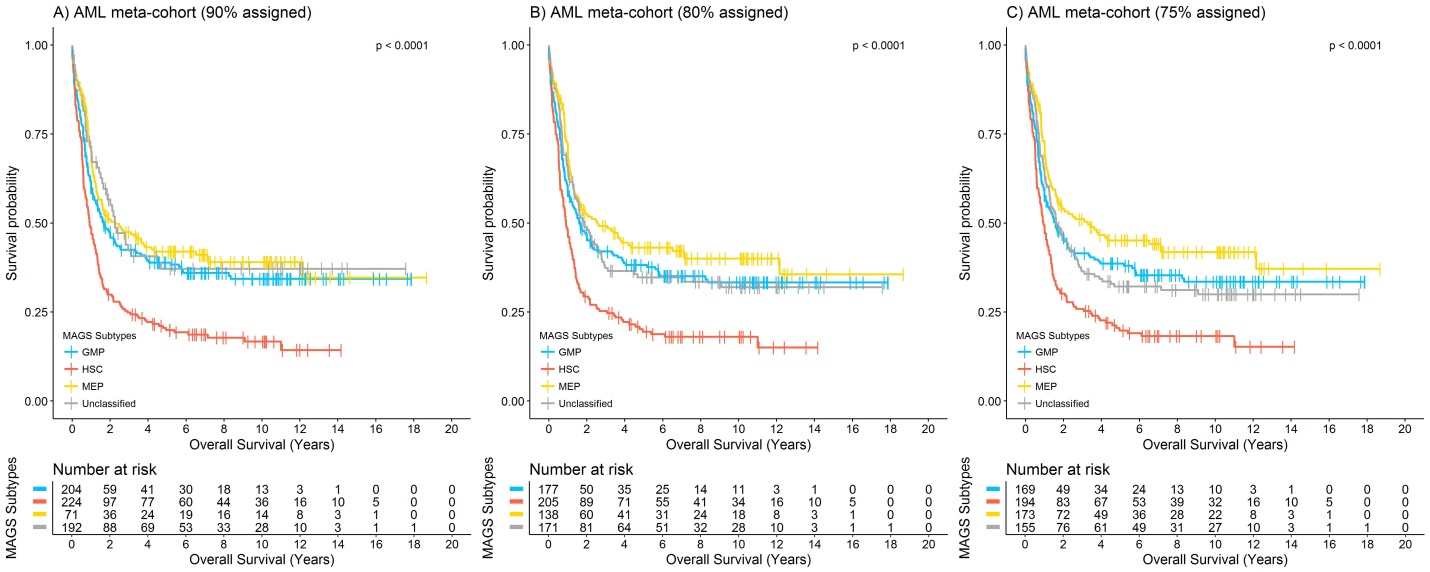

Supplement: S4 Fig — (DOCX) [file pone.0229593.s014.docx]
